# Supplementary material for: SAM68 directs STING signaling to apoptosis in macrophages
Source: Commun Biol. 2024 Mar 7;7:283. doi: 10.1038/s42003-024-05969-1 (PMC10920828; doi:10.1038/s42003-024-05969-1)
Supplement: Supplementary file 2 — Supplemental Material [file 42003_2024_5969_MOESM2_ESM.pdf]

Supplemental information

SAM68 directs STING signaling to apoptosis in macrophages

Demi van der Horst[1], Naziia Kurmasheva[*1], Mikkel H.S. Marqvorsen[*1], Sonia Assil[*1], Anna H. F. Rahimic[1], Christoph F. Kollmann[1], Leandro Silva da Costa[1], Qi Wu[1], Jian Zhao[1], Eleonora Cesari[2], Marie B. Iversen[1], Fanghui Ren[1], Trine I. Jensen[1], Ryo Narita[1], Vivien R. Schack[1], Bao-cun Zhang[1], Rasmus O. Bak[1], Claudio Sette[2,3], Robert A Fenton[1], Jacob G. Mikkelsen[1], Søren R. Paludan[**#1], David Olagnier[**#1]

[1] Department of Biomedicine, Aarhus University, Høegh Guldbergsgade 10, 8000 Aarhus C, Denmark

[2] GSTEP-Organoids Core Facility, IRCCS Fondazione Policlinico Agostino Gemelli, 00168 Rome, Italy

[3] Department of Neuroscience, Section of Human Anatomy, Catholic University of the Sacred Hearth, 00168 Rome, Italy

Short title: STING engages SAM68 to induce apoptosis

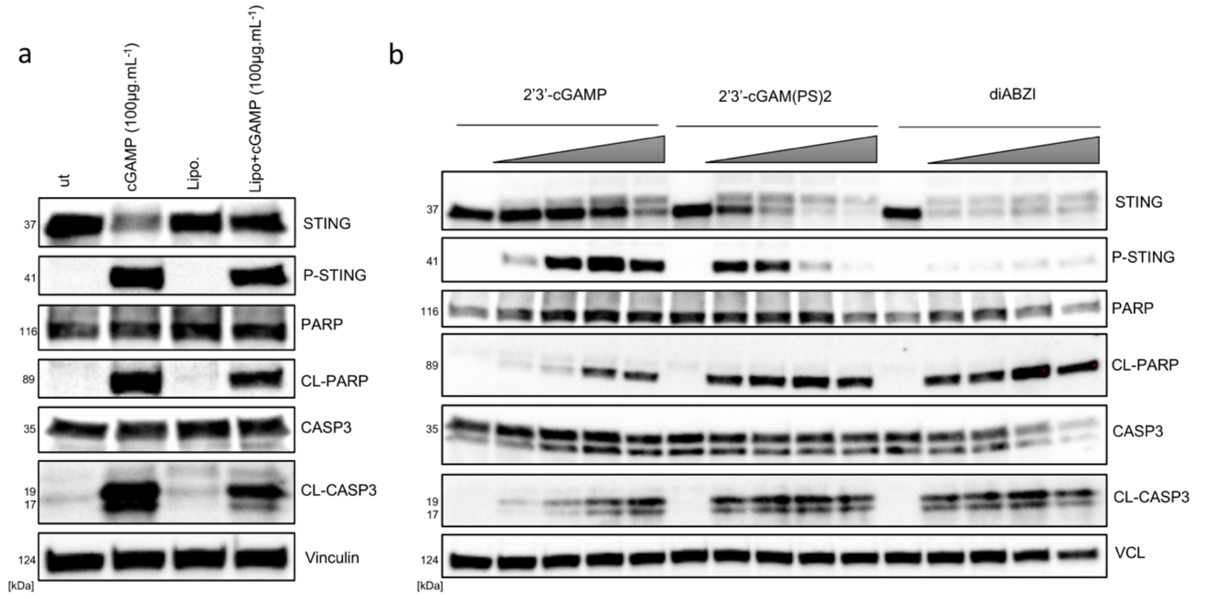

Figure S1. STING induction induces cleavage of caspase 3 and PARP in PMA-THP1

(**a**) PMA-differentiated THP1 cells (PMA-THP1) were treated with cGAMP exogenously (100 µg.mL^{-1}) or lipofected with cGAMP (10 µg.mL^{-1}) for 5h and lysates were immunoblotted for cleaved (CL) caspase 3, total caspase 3, cleaved PARP, total PARP, phospho-STING (P-STING), total STING and vinculin (n=2).

(**b**) PMA-differentiated THP1 cells (PMA-THP1) were treated with 2’3’-cGAMP, 2’3’-cGAM(PS)$_2$, or diABZI exogenously (at 12.5, 25, 50 or 100 µg.mL^{-1}) for 5h and cell lysates were immunoblotted for cleaved (CL) caspase 3, total caspase 3, cleaved PARP, total PARP, phospho-STING (P-STING), total STING and vinculin (n=2)

Vertical stacks of bands are not derived from the same membrane in (a) and (b).

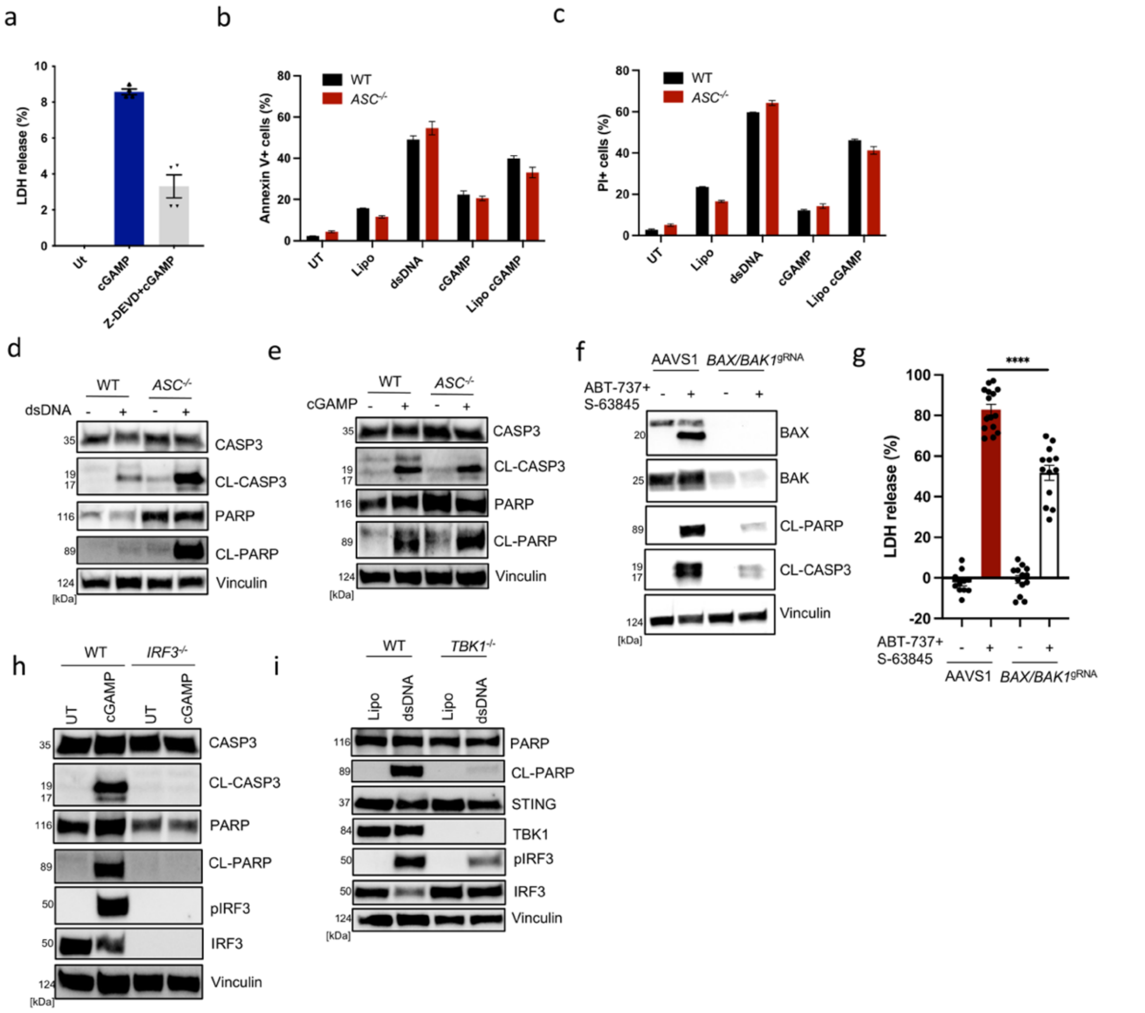

Figure S2. STING induces cell death through the mitochondrial apoptosis pathway in monocytes. (**a**) PMA-differentiated THP1 cells (PMA-THP1) were treated with Z-DEVD (25µM) 1 h prior to treatment with cGAMP (100 µg.mL^{-1}). LDH levels in the culture supernatants isolated 8h later were measured by LDH release assay. Data are the means ± SEM of two independent experiments performed in duplicates.

(**b-c**) WT and *ASC*$^{-/-}$ THP1 cells were treated with dsDNA (4µg.mL^{-1}) or cGAMP (100 µg.mL^{-1}) for 20h, and evaluated for staining for Annexin V and Propidium Iodide (PI) by flow cytometry (n=2). Data are the means ± SEM of two independent experiments performed in duplicates.

(**d-e**) WT and *ASC*$^{-/-}$ PMA-THP1 cells were treated with dsDNA (4µg.mL^{-1}) or cGAMP (100 µg.mL^{-1}) for 5h, and lysates were immunoblotted for Cleaved (CL) caspase 3, total caspase 3, cleaved PARP, total PARP and vinculin.

(**f-g**) THP1 cells were treated with Cas9-gRNA RNP complexes targeting AAVS1 or a combination of BAX and BAK1 gRNA and treated with a combination of ABT-737 and S-63845 (5 µg.mL^{-1} each drug) for 5h in (f) and 19h in (g). Cell survival/apoptosis induction was monitored by LDH release assay and immunoblotting for cleavage of Caspase 3 and PARP, respectively. Data are the means of 2 independent experiments ± SEM performed in several biological replicates (g). Data are from one representative experiment performed twice in (f).

(h-i) WT*, IRF3*$^{-/-}$*, and TBK1*$^{-/-}$, PMA-THP1 cells were treated with dsDNA (4µg.mL^{-1}) or cGAMP (100 µg.mL^{-1}) for 5h, and lysates were immunoblotted for the indicated protein (n=more than 3 for most of the KO cell lines tested). CL, cleaved. pSTING, phosphorylation at S366; pTBK1, phosphorylation as S172; pIRF3, phosphorylation at S396.

Statistical analysis of the data in (g) was performed using a two-tailed one-way ANOVA followed by Sidak's multiple comparison test. Vertical stacks of bands are not derived from the same membrane in (d), (e), (f), (h) and (i).

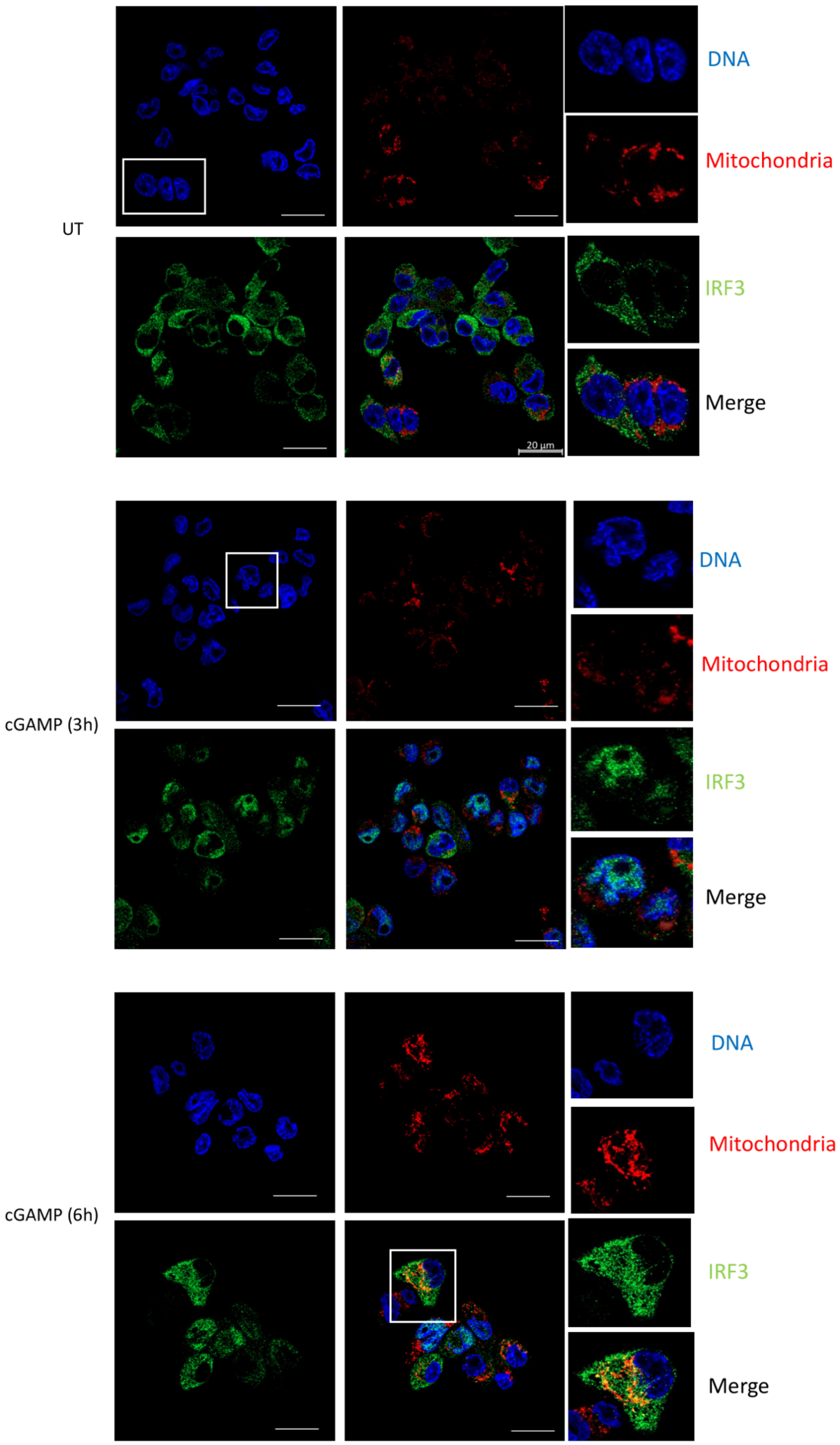
UT
DNA
Mitochondria
IRF3
Merge
20 µm
cGAMP (3h)
DNA
Mitochondria
IRF3
Merge
cGAMP (6h)
DNA
Mitochondria
IRF3
Merge

Figure on previous page

Figure S3. **IRF3 localizes to the mitochondria following cGAMP stimulation in PMA-THP1**
PMA-THP1 cells were treated with cGAMP (100μg.mL^{-1}) for the indicated period of times, and stained for DNA, IRF3 and mitochondria. Cells were visualized by confocal microscopy (n=3). Scale bars, 20 μm

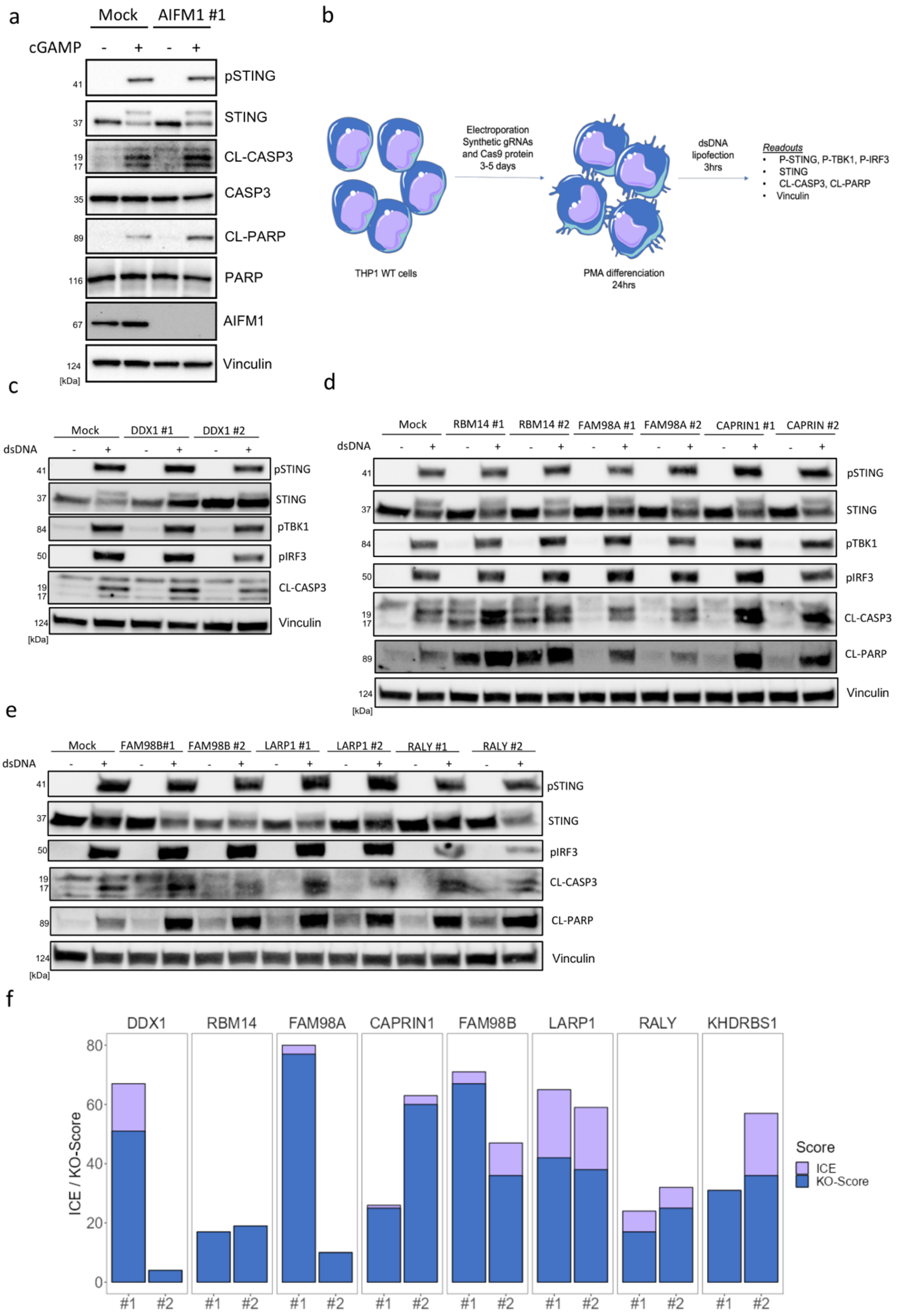
a
Mock
AIFM1 #1
cGAMP
pSTING
STING
CL-CASP3
CASP3
CL-PARP
PARP
AIFM1
Vinculin
[kDa]
b
Electroporation
Synthetic gRNAs
and Cas9 protein
3-5 days
THP1 WT cells
PMA differenciation
24hrs
dsDNA
lipofection
3hrs
Readouts
P-STING, P-TBK1, P-IRF3
STING
CL-CASP3, CL-PARP
Vinculin
c
Mock
DDX1 #1
DDX1 #2
dsDNA
pSTING
STING
pTBK1
pIRF3
CL-CASP3
Vinculin
d
Mock
RBM14 #1
RBM14 #2
FAM98A #1
FAM98A #2
CAPRIN1 #1
CAPRIN #2
dsDNA
pSTING
STING
pTBK1
pIRF3
CL-CASP3
CL-PARP
Vinculin
e
Mock
FAM98B#1
FAM98B #2
LARP1 #1
LARP1 #2
RALY #1
RALY #2
dsDNA
pSTING
STING
pIRF3
CL-CASP3
CL-PARP
Vinculin
f
DDX1
RBM14
FAM98A
CAPRIN1
FAM98B
LARP1
RALY
KHDRBS1
ICE / KO-Score
Score
ICE
KO-Score

Figure on previous page

Figure S4. Identification of proteins involved in STING-induced apoptosis.

(a) THP1 cells were treated with gRNA/Cas9 RNP complexes targeting the AIFM1 gene and differentiated into PMA-THP1 macrophages. The cells were treated with cGAMP (100µg.mL^{-1}) for 5h and lysates were immunoblotted as shown (n=2). CL, cleaved. pSTING, phosphorylation at S366.

(**b**) Set-up for analysis of identified candidates.

(**c-e**) THP1 cells were treated with gRNA/Cas9 RNP complexes targeting the indicated genes (two gRNAs per gene), and differentiated into PMA-THP1 macrophages. The cells were treated with dsDNA (4µg.mL^{-1}) for 5h and lysates were immunoblotted as shown (n=2). CL, cleaved. pSTING, phosphorylation at S366; pTBK1, phosphorylation at S172; pIRF3, phosphorylation at S396.

(f) Indel and KO rates of all guide RNA sequences used in the study. THP1 cells were electroporated with gRNA in presence of Cas9 protein. 72 hours post electroporation cells were analyzed for KO efficiency by sequencing and genomic analysis. Two different gRNA sequences have been used for each gene tested.

Vertical stacks of bands are not derived from the same membrane in (a), (c), (d) and (e).

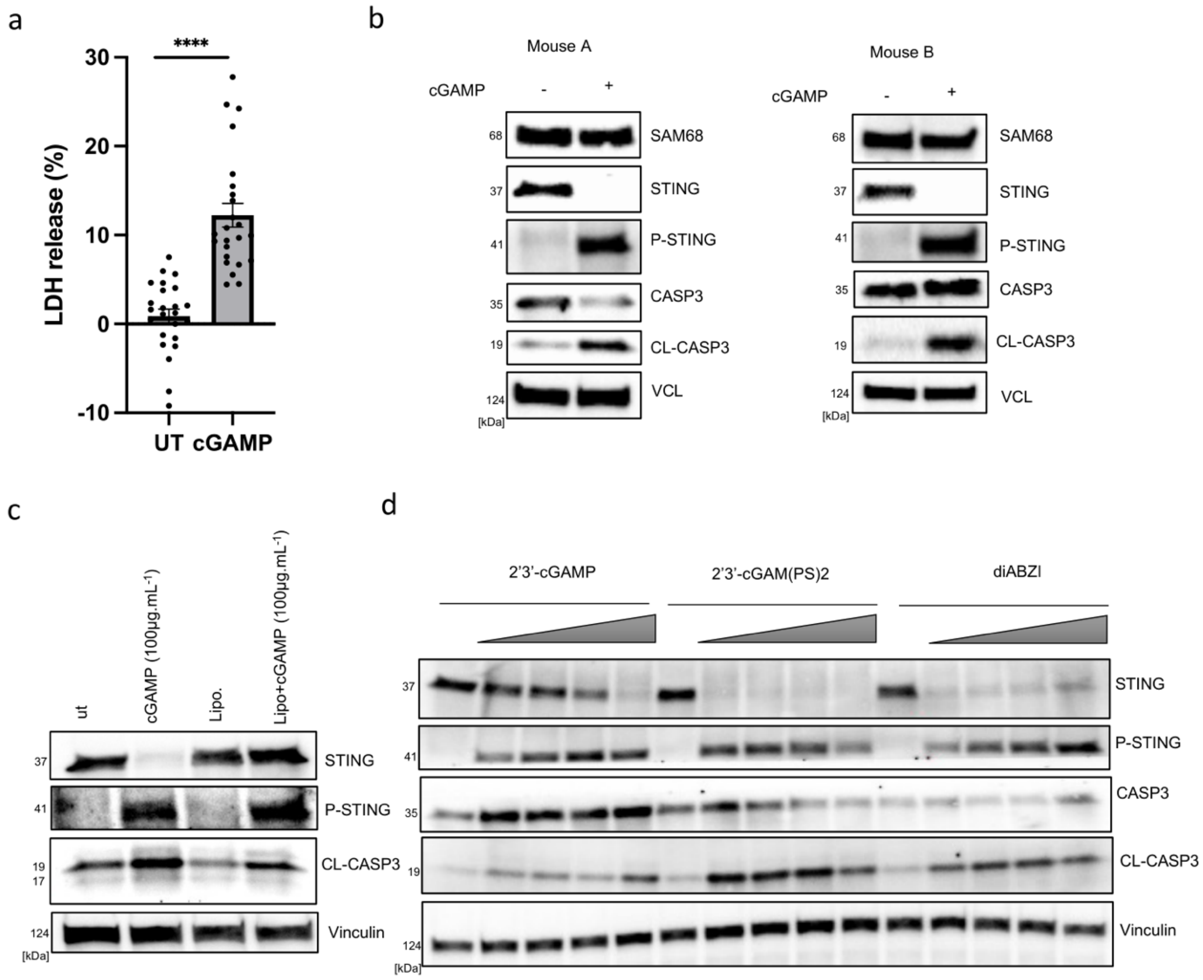

Figure S5. cGAMP induces cell death and cleavage of Caspase 3 in murine BMDMs.

(a) Bone marrow-derived macrophages (BMDMs) were treated with cGAMP (100 µg.mL^{-1}). LDH levels in the culture supernatants was measured 20h post cGAMP stimulation by LDH release assay (n=3 from cells differentiated from different animals). Data are the means ± SEM of three independent experiments performed in several replicates.

(**b**) BMDMs were treated with cGAMP (100 µg.mL^{-1}) for 5h, and lysates were immunoblotted for the indicated proteins. Data are shown from BMDMs derived from two different animals. Experiment was performed once.

(c) Bone marrow-derived macrophages (BMDMs) were treated with cGAMP exogenously (100 µg.mL^{-1}) or lipofected with cGAMP (10 µg.mL^{-1}). for 5h and cell lysates were immunoblotted for the indicated proteins. This immunoblot is representative of two immunoblots performed on cells differentiated from 2 different animals.

(**d**) BMDMs were treated with 2'3'-cGAMP, 2'3'-cGAM(PS)$_2$, or diABZI exogenously (at 12.5, 25, 50 or 100 µg.mL^{-1}) for 5h and cell lysates were immunoblotted for the indicated proteins. This immunoblot is representative of four immunoblots performed on cells differentiated from 4 different animals.Statistical analysis of the data in (a) was performed using a Student's t-test.Vertical stacks of bands are not derived from the same membrane in (b), (c) and (d).

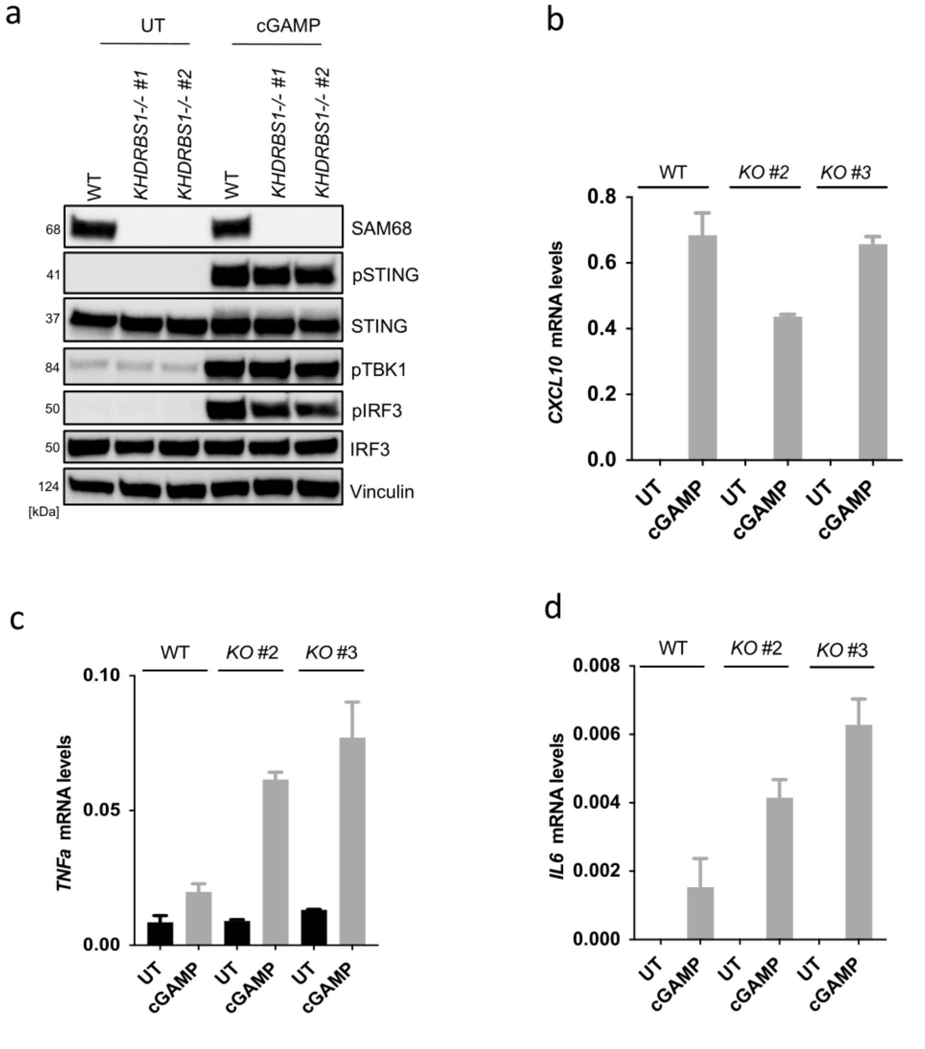

Figure S6. SAM68 is not involved in STING-mediated activation of the IFN and NF-κB responses.

(**a**) WT and *KHDRBS1*$^{-/-}$ PMA-THP1 cells were treated with cGAMP (100µg.mL^{-1}) for 5h, and lysates were immunoblotted for the indicated proteins (n=3). pSTING, phosphorylation at S366; pTBK1, phosphorylation as S172; pIRF3, phosphorylation at S396.

(**b-d**) WT and *KHDRBS1*$^{-/-}$ PMA-THP1 cells were treated with cGAMP (100µg.mL^{-1}) for 5h. RNA was isolated and examined for levels of *CXCL10*, *TNFA*, and *IL6*. Data are means for one experiment performed in biological duplicates.

Vertical stacks of bands are not derived from the same membrane in (a).

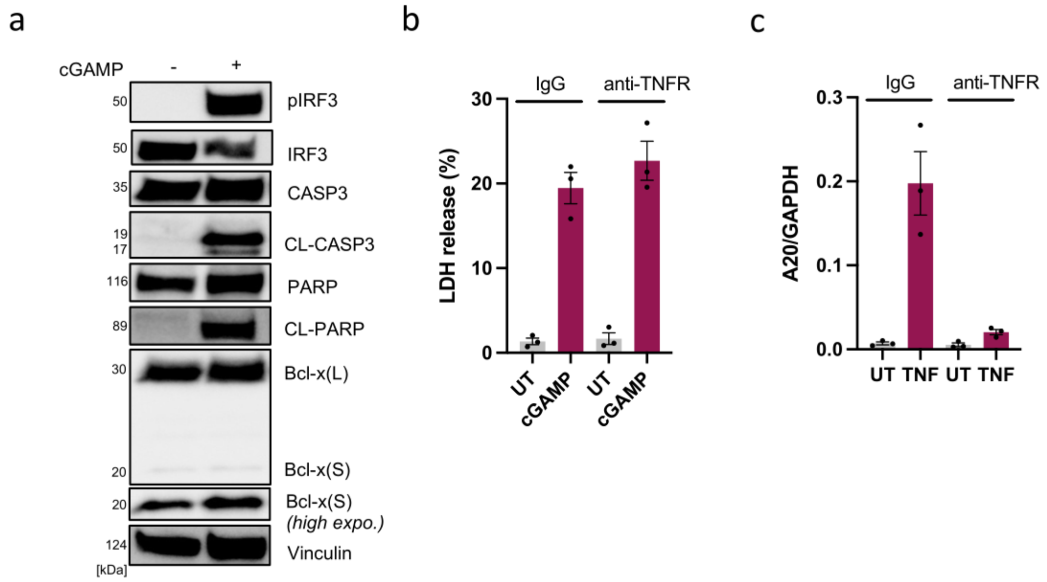

Figure S7. SAM68 does not promote STING-dependent apoptosis through known mechanisms.

(**a**) PMA-THP1 cells were treated with cGAMP (100µg.mL^{-1}) for 5h, and lysates were immunoblotted for the indicated proteins (n=2). CL, cleaved. pIRF3, phosphorylation at S396.

(**b-c**) PMA-THP1 cells were treated with neutralizing Rabbit anti-human TNF-α IgG or control Rabbit IgG (10 µg/ml), 30 min before stimulation with cGAMP (100µg.mL^{-1}) or TNF-α (20 ng/ml). Culture supernatants were collected 16h after cGAMP stimulation, and total RNA was isolated 4h after TNF-α stimulation and LDH release and A20 mRNA levels were measured, respectively (n=2). Data are the means ± SEM of three independent experiments.
Vertical stacks of bands are not derived from the same membrane in (a).

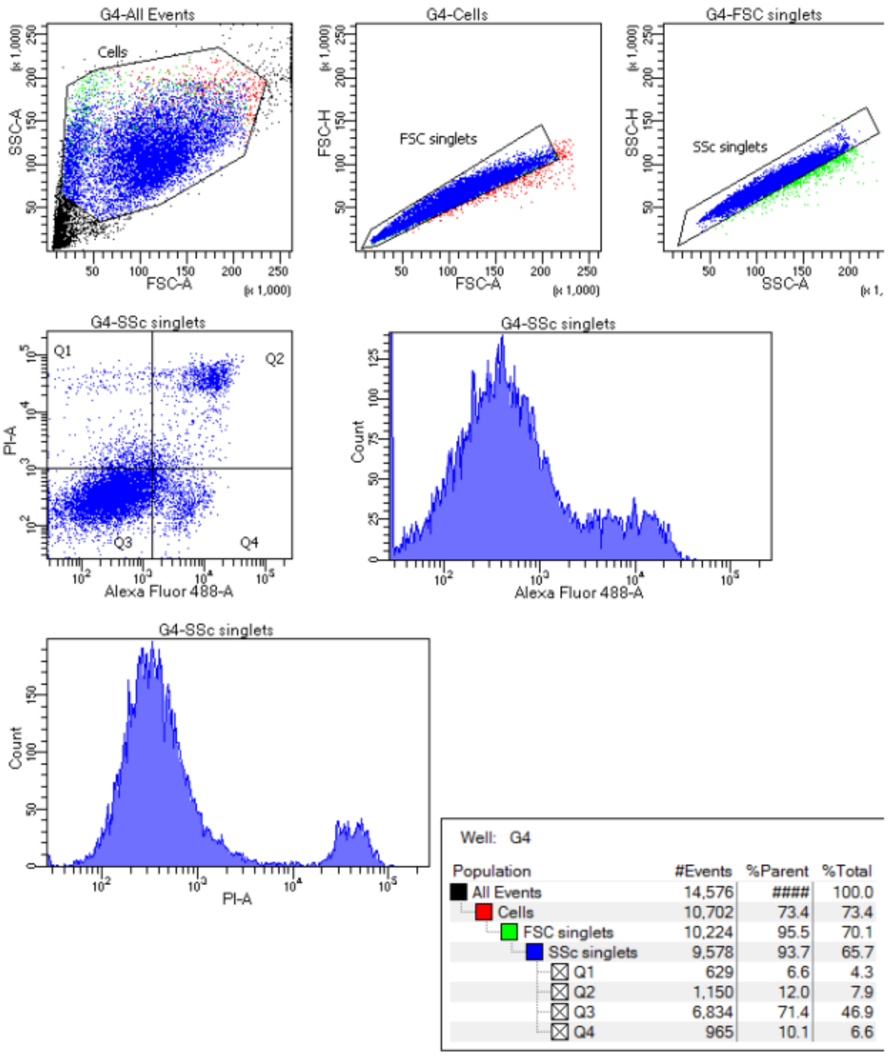

Figure S8. Gating strategy for flow cytometry

Gating strategy for flow cytometric assessment of cell death/apoptosis (Annexin V-Alexa Fluor 488 / PI).

Figure S9. Uncropped images of the western blots presented in the manuscript.

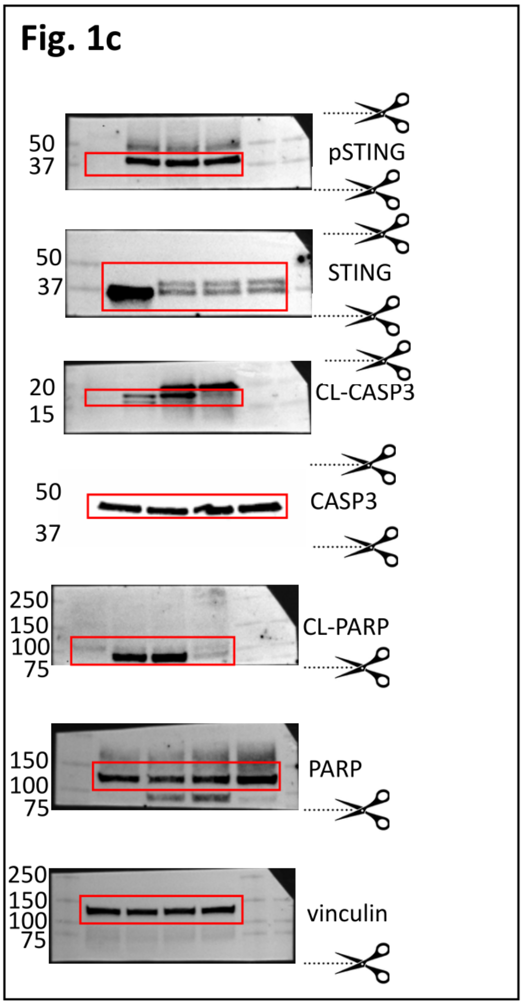

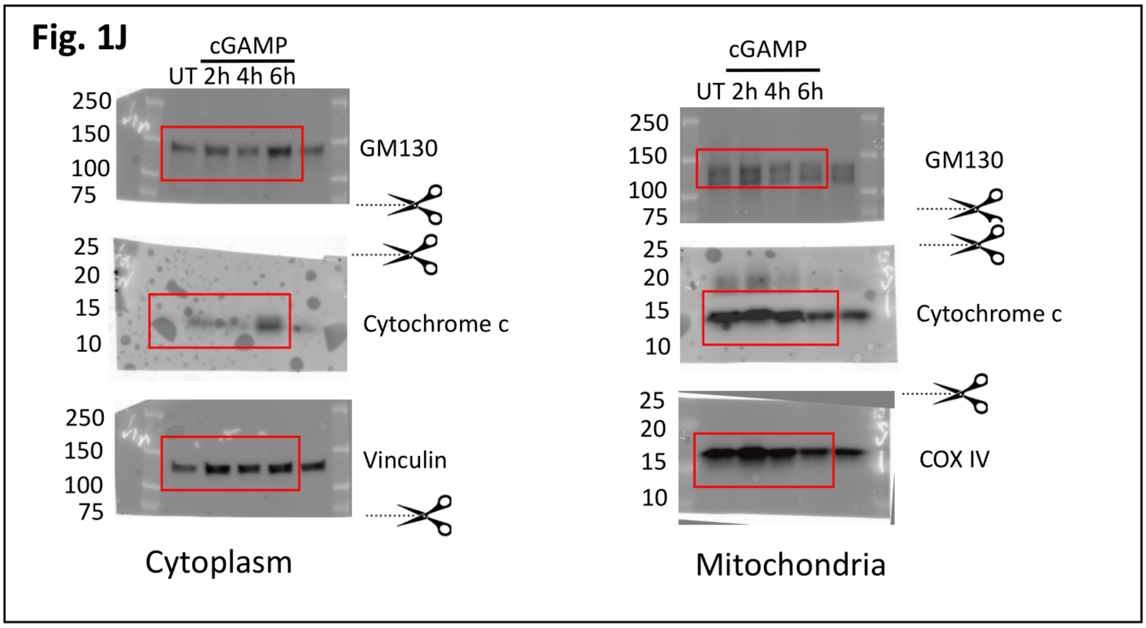

Fig. 1k

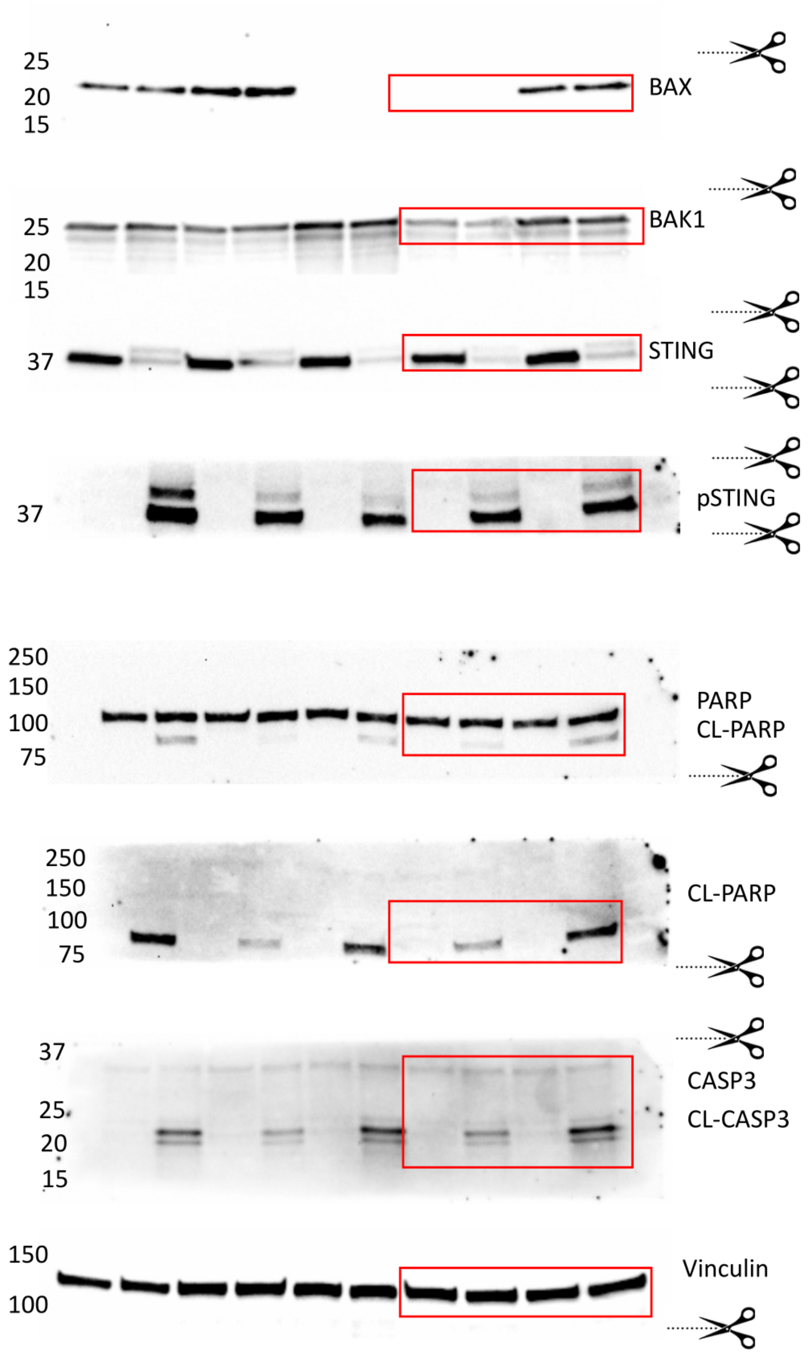
25
20
15
BAX
25
20
15
BAK1
37
STING
37
pSTING
250
150
100
75
PARP
CL-PARP
250
150
100
75
CL-PARP
37
25
20
15
CASP3
CL-CASP3
150
100
Vinculin

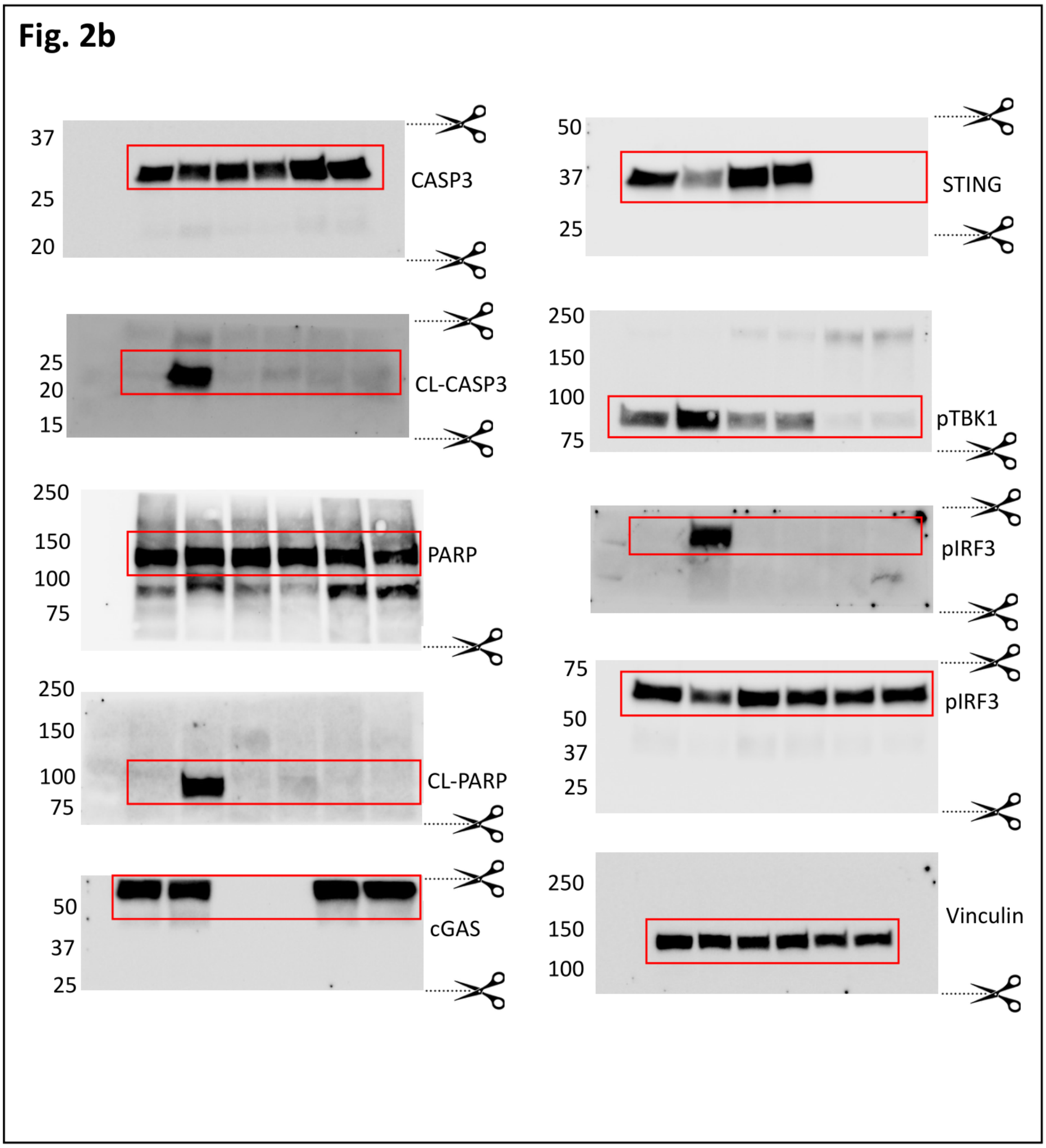
Fig. 2b
37
25
20
CASP3
50
37
25
STING
25
20
15
CL-CASP3
250
150
100
75
pTBK1
250
150
100
75
PARP
pIRF3
75
50
37
25
pIRF3
250
150
100
75
CL-PARP
50
37
25
cGAS
250
150
100
Vinculin

Fig. 2c

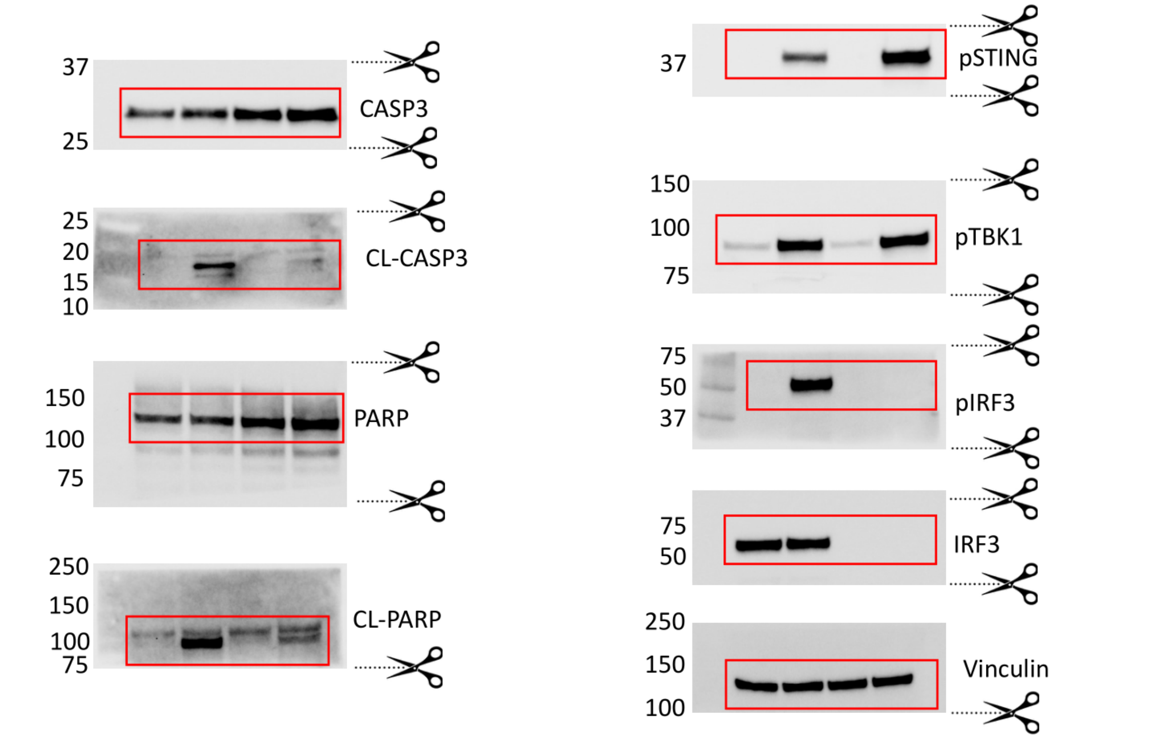

Fig. 2d

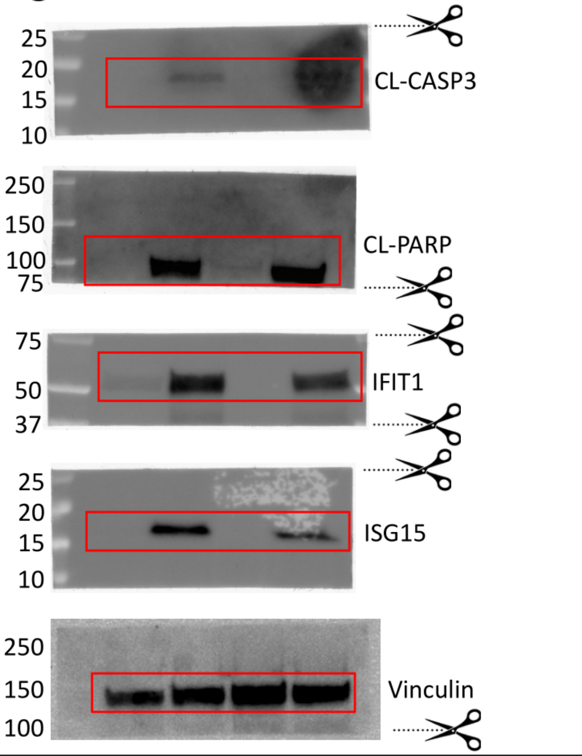

Fig. 2e

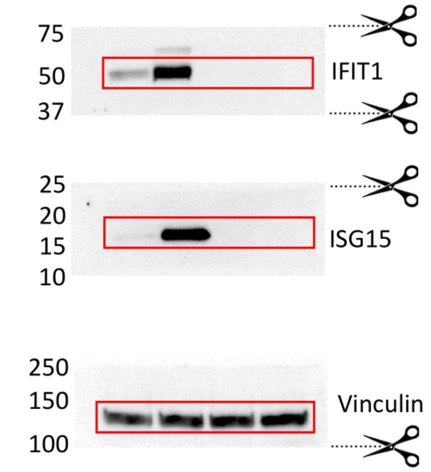

Fig. 2f

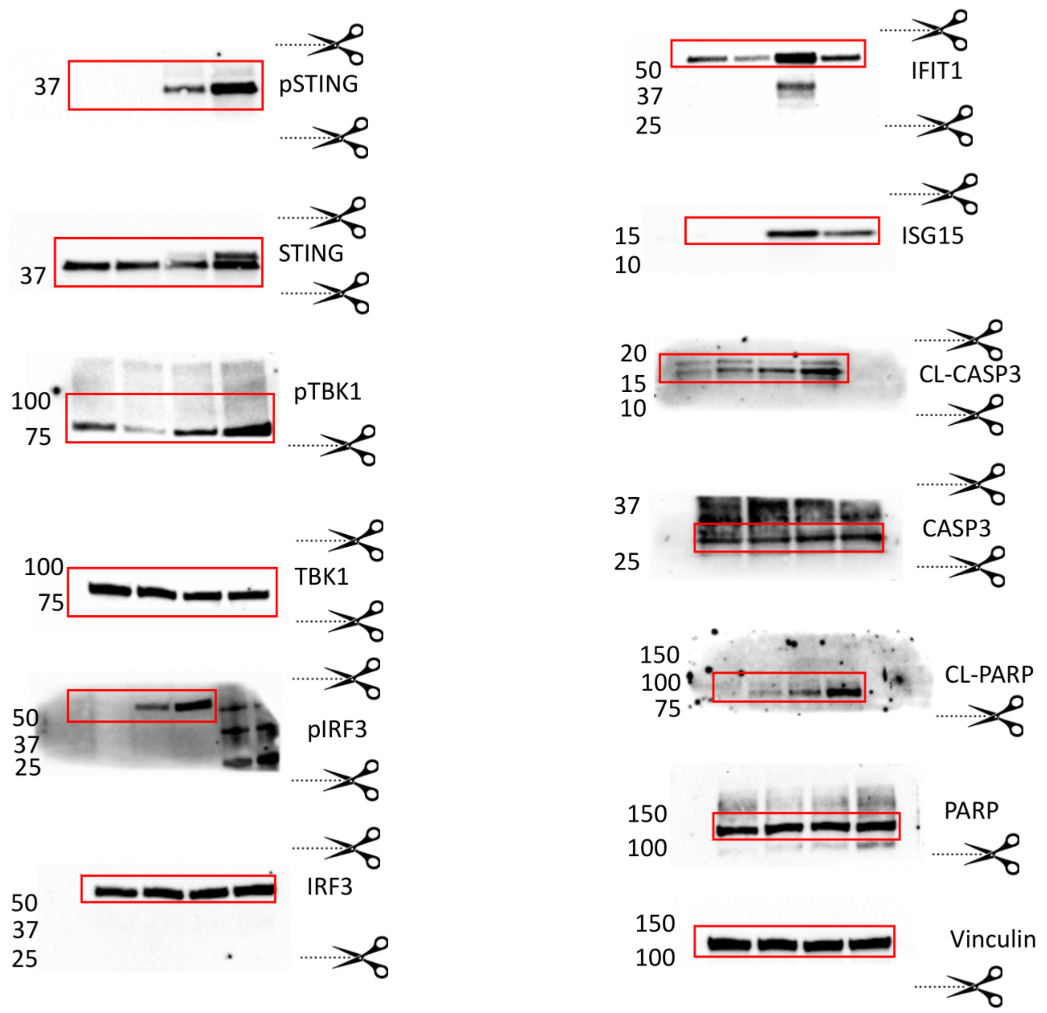

37
pSTING
37
STING
100
75
pTBK1
100
75
TBK1
50
37
25
pIRF3
50
37
25
IRF3
50
37
25
IFIT1
15
10
ISG15
20
15
10
CL-CASP3
37
25
CASP3
150
100
75
CL-PARP
150
100
PARP
150
100
Vinculin

Fig. 2g

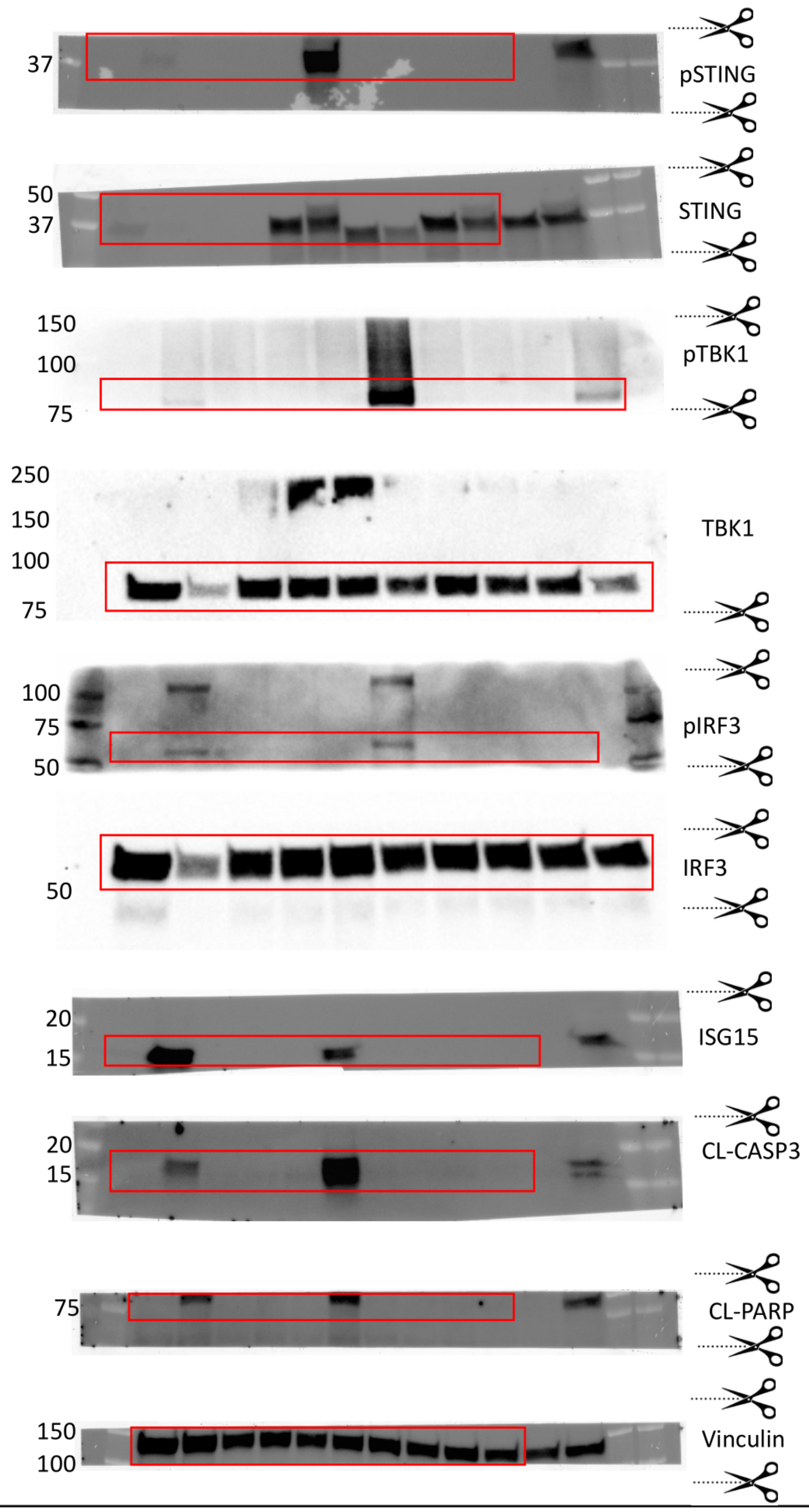
37
pSTING
50
37
STING
150
100
75
pTBK1
250
150
100
75
TBK1
100
75
50
pIRF3
50
IRF3
20
15
ISG15
20
15
CL-CASP3
75
CL-PARP
150
100
Vinculin

Fig. 3c

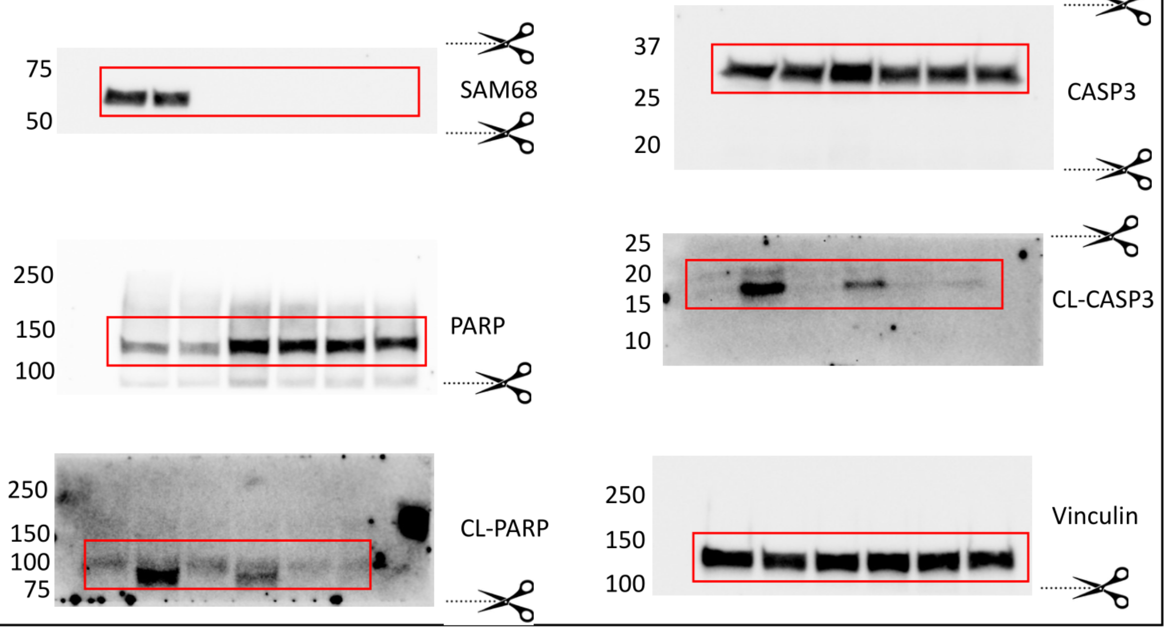
75
50
SAM68
37
25
20
CASP3
250
150
100
PARP
25
20
15
10
CL-CASP3
250
150
100
75
CL-PARP
250
150
100
Vinculin

Fig. 3d

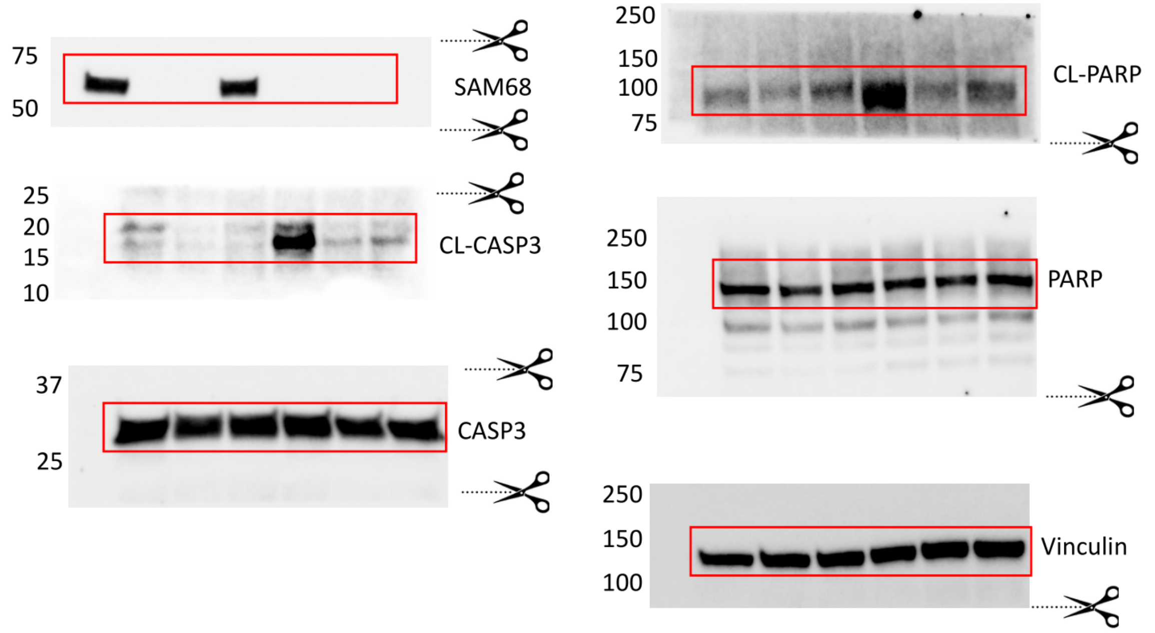
75
50
SAM68
250
150
100
75
CL-PARP
25
20
15
10
CL-CASP3
250
150
100
75
PARP
37
25
CASP3
250
150
100
Vinculin

Fig. 3i

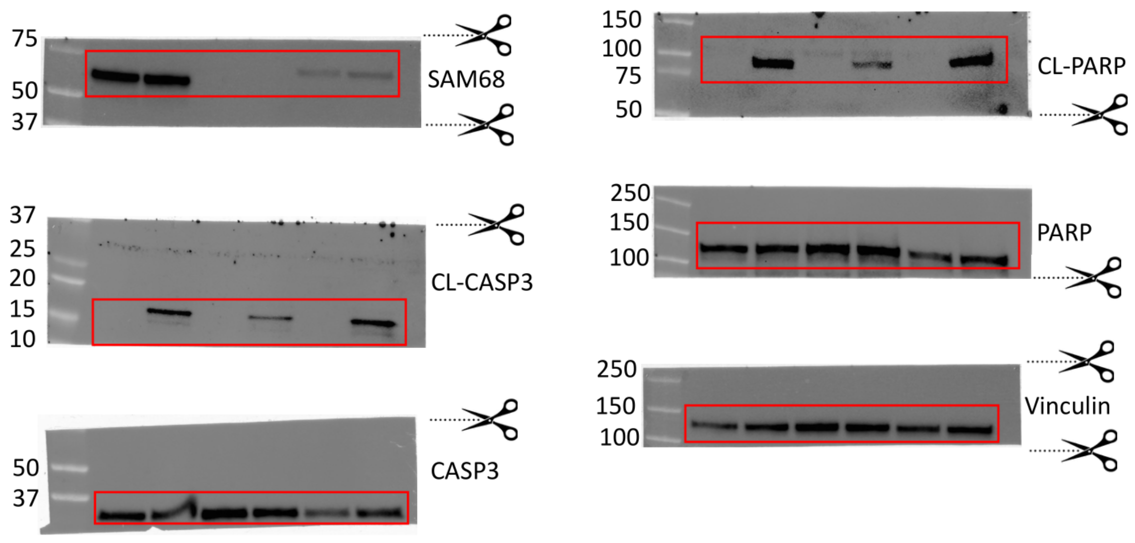

Fig. 3j

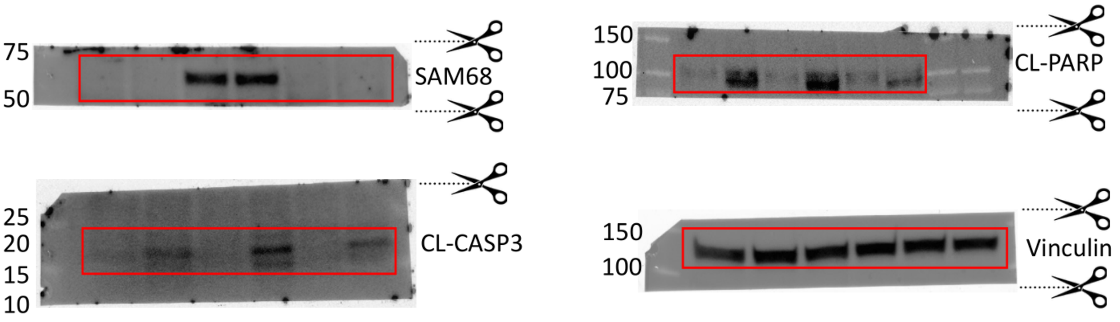

Fig. 3l

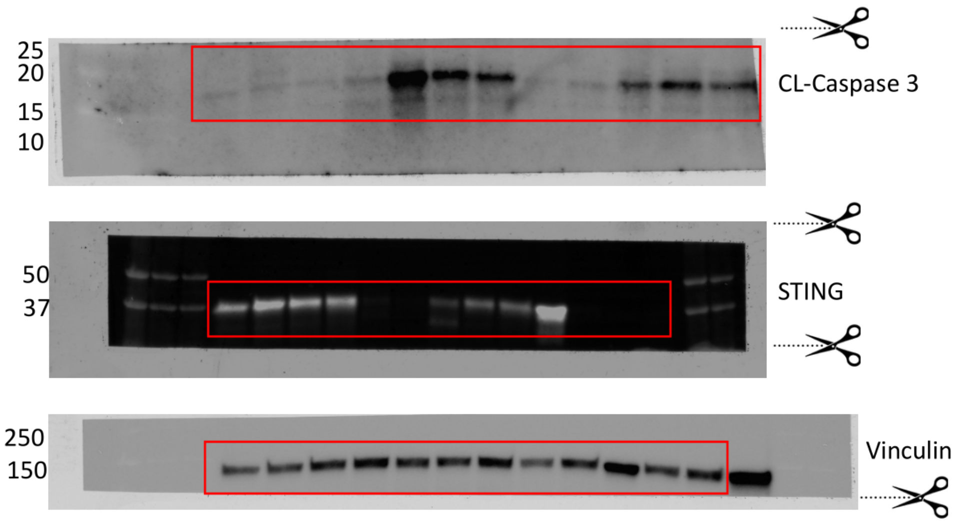

Fig. 4b

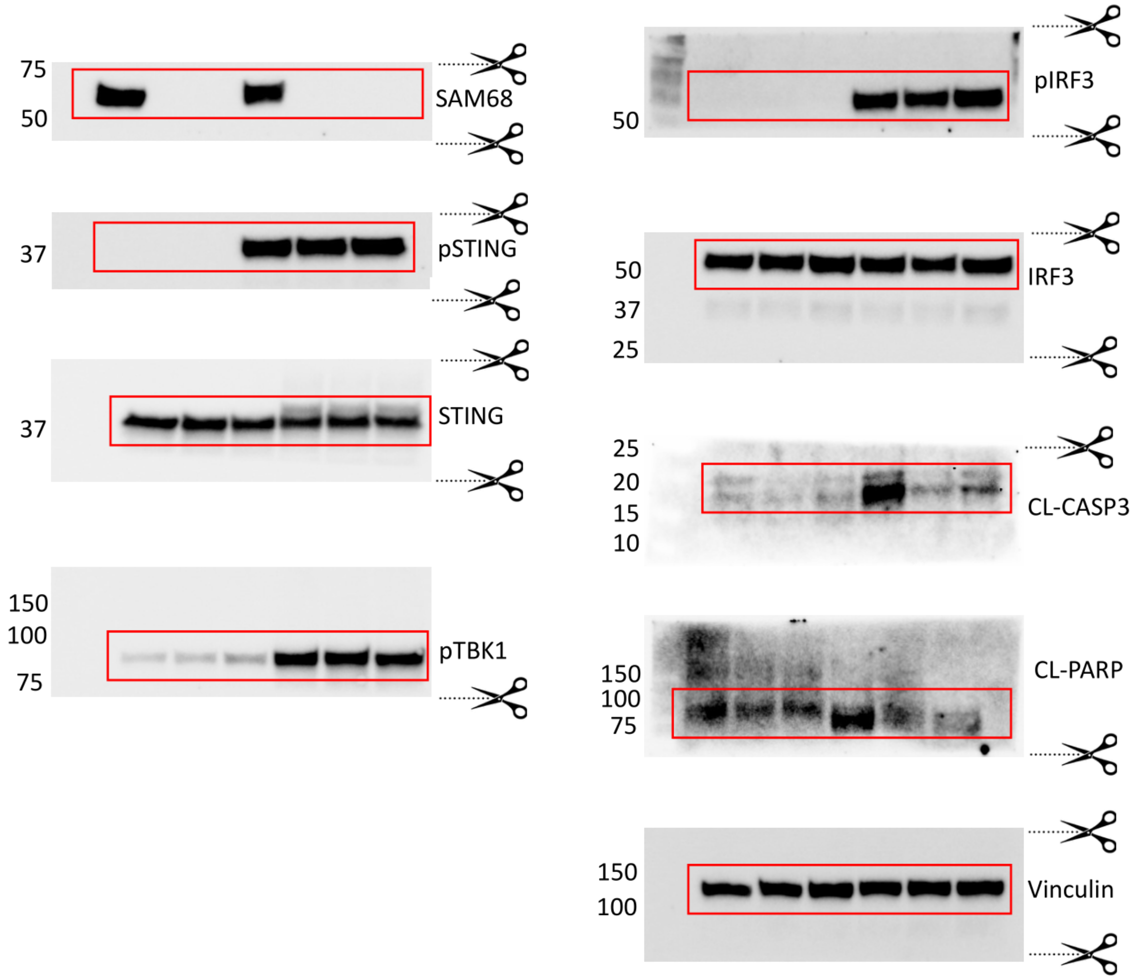
75
50
SAM68
37
pSTING
37
STING
150
100
75
pTBK1
50
pIRF3
50
37
25
IRF3
25
20
15
10
CL-CASP3
150
100
75
CL-PARP
150
100
Vinculin

Fig. 4c

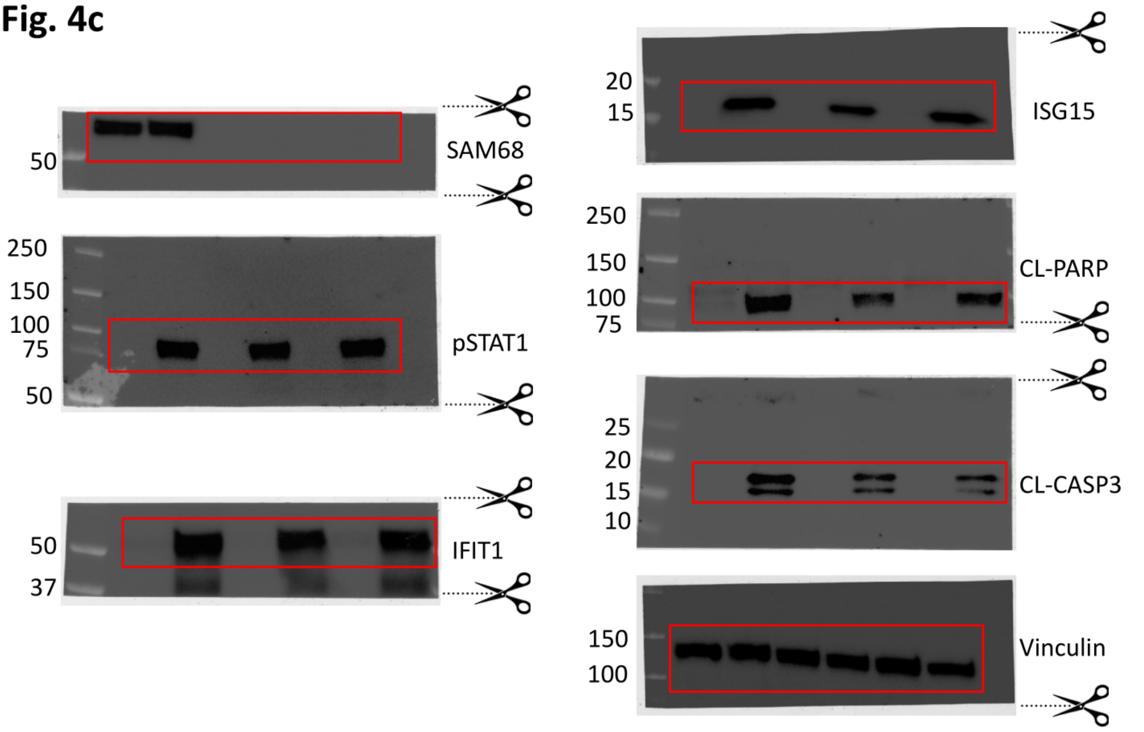
SAM68
50
250
150
100
75
50
pSTAT1
50
37
IFIT1
20
15
ISG15
250
150
100
75
CL-PARP
25
20
15
10
CL-CASP3
150
100
Vinculin

Fig. 5a

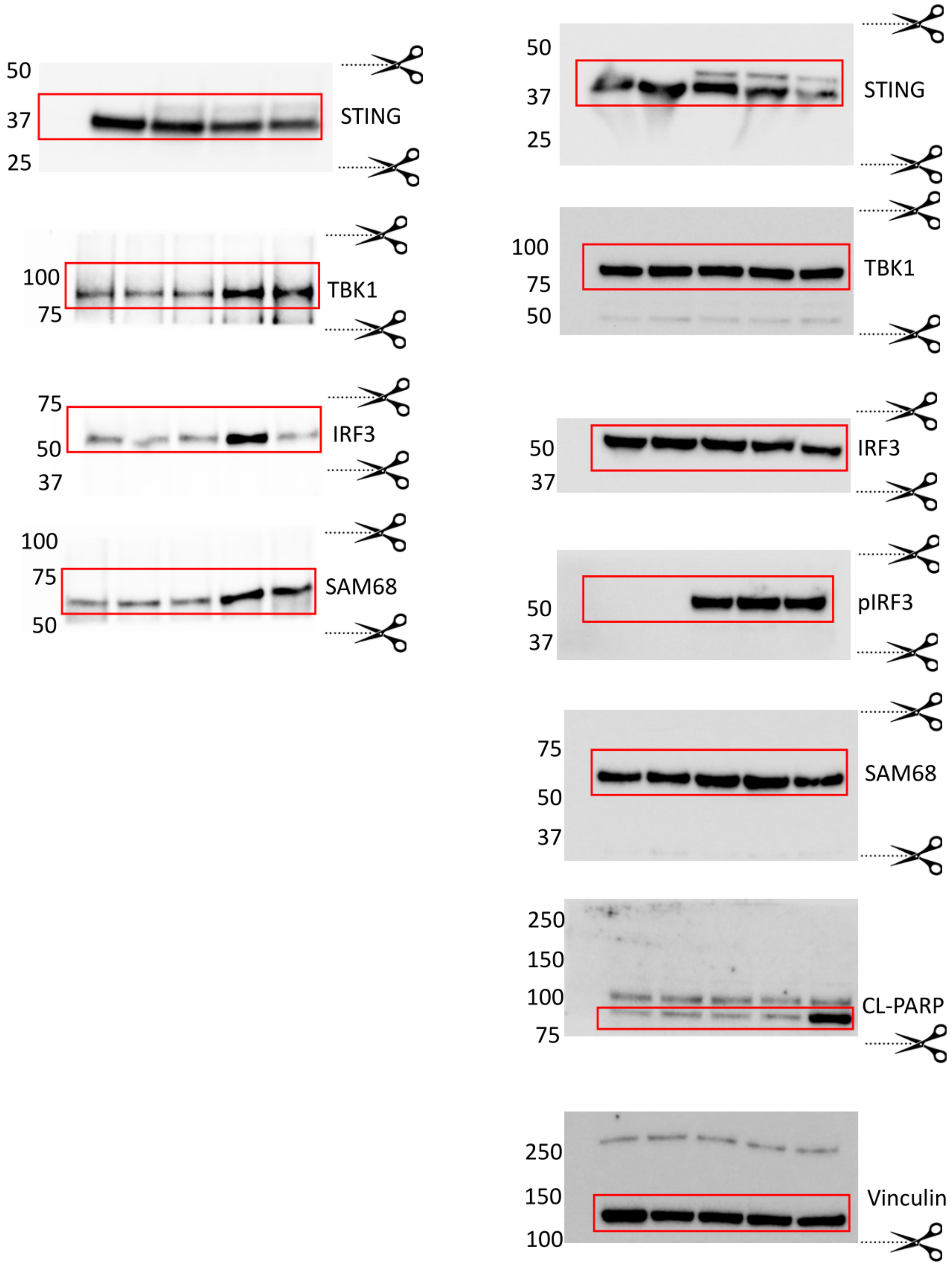
50
37
25
STING
100
75
TBK1
75
50
37
IRF3
100
75
50
SAM68
50
37
25
STING
100
75
50
TBK1
50
37
IRF3
50
37
pIRF3
75
50
37
SAM68
250
150
100
75
CL-PARP
250
150
100
Vinculin

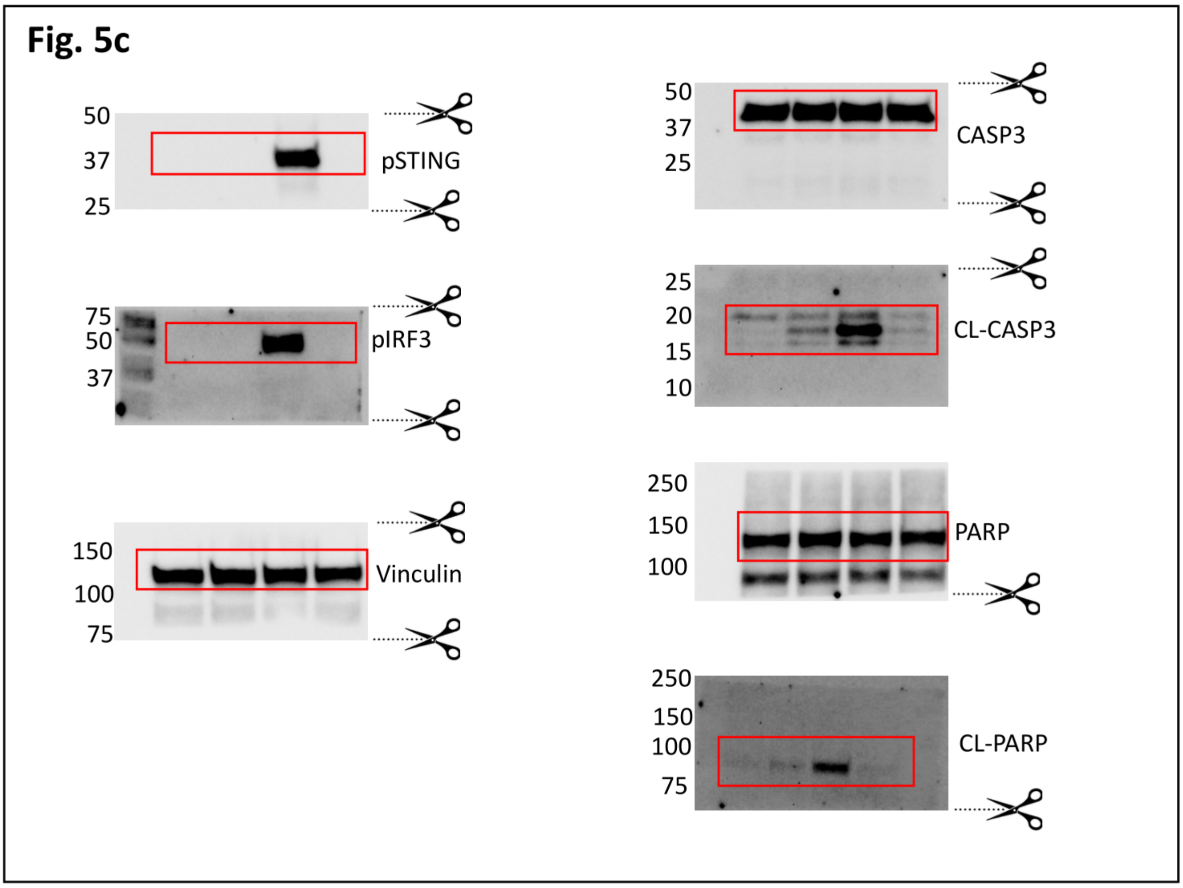

Fig. 5c
50
37
25
pSTING
75
50
37
pIRF3
150
100
75
Vinculin
50
37
25
CASP3
25
20
15
10
CL-CASP3
250
150
100
PARP
250
150
100
75
CL-PARP

Fig. 5d

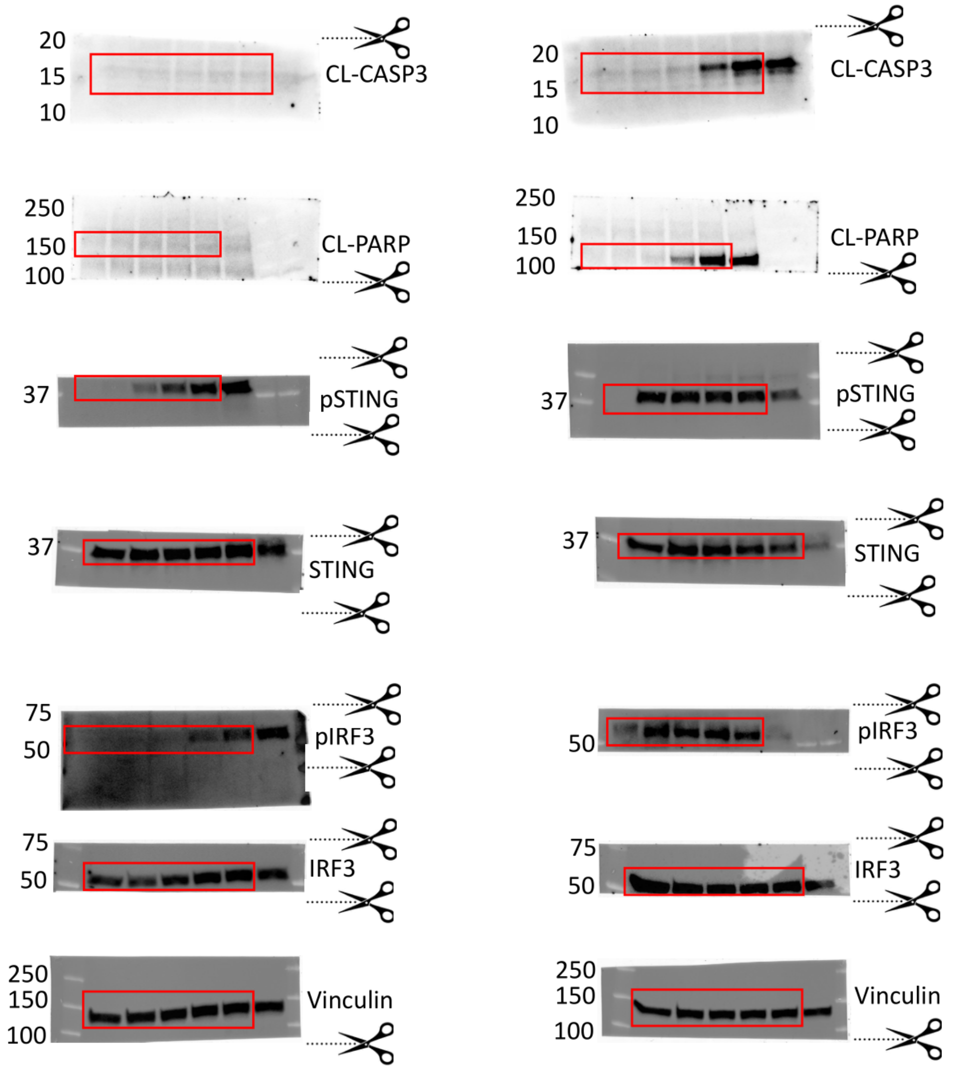

20
15
10
CL-CASP3
250
150
100
CL-PARP
37
pSTING
37
STING
75
50
pIRF3
75
50
IRF3
250
150
100
Vinculin
20
15
10
CL-CASP3
250
150
100
CL-PARP
37
pSTING
37
STING
50
pIRF3
75
50
IRF3
250
150
100
Vinculin

Fig. 5e

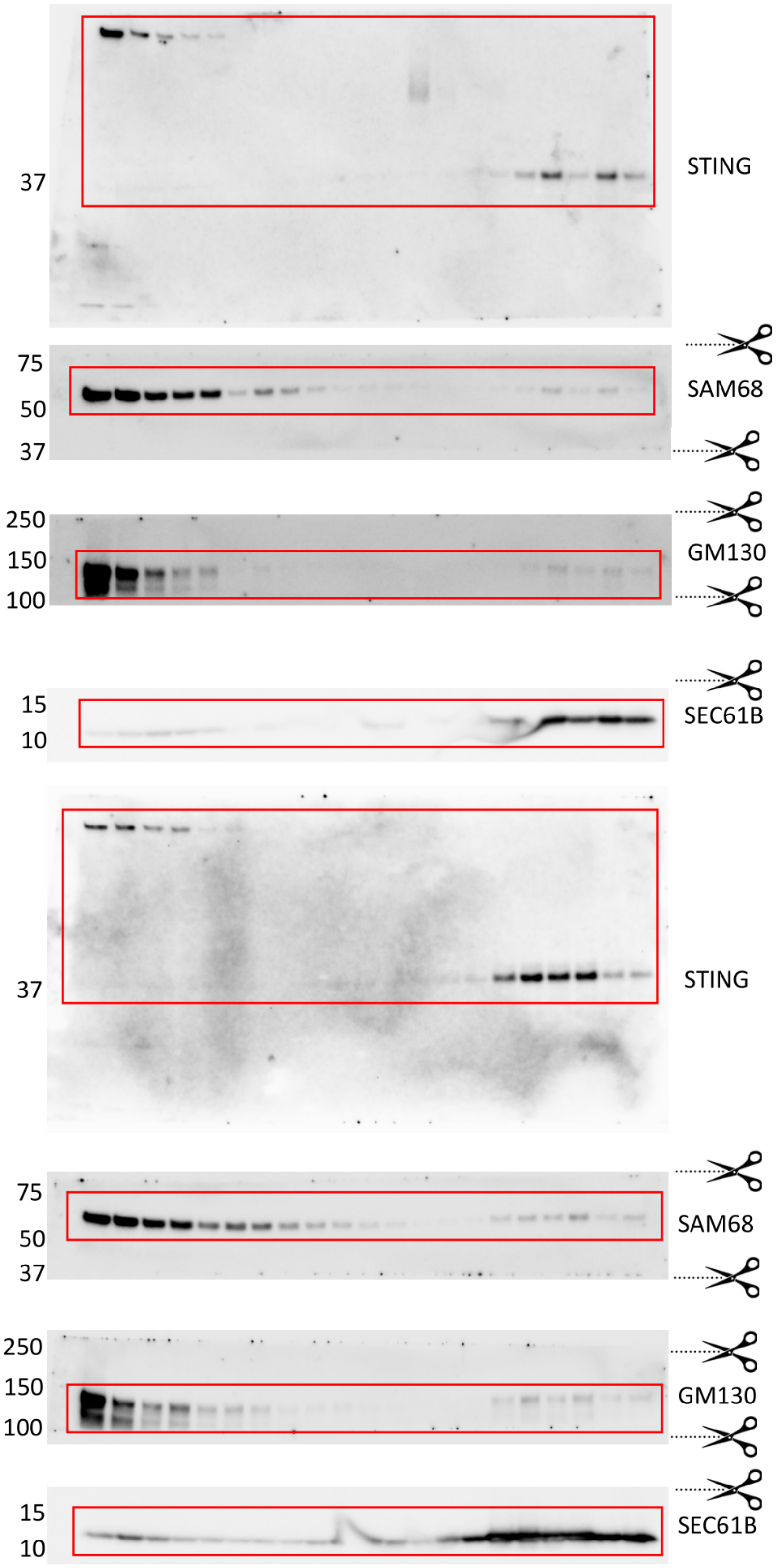
37
STING
75
50
37
SAM68
250
150
100
GM130
15
10
SEC61B
37
STING
75
50
37
SAM68
250
150
100
GM130
15
10
SEC61B

Fig. S1a

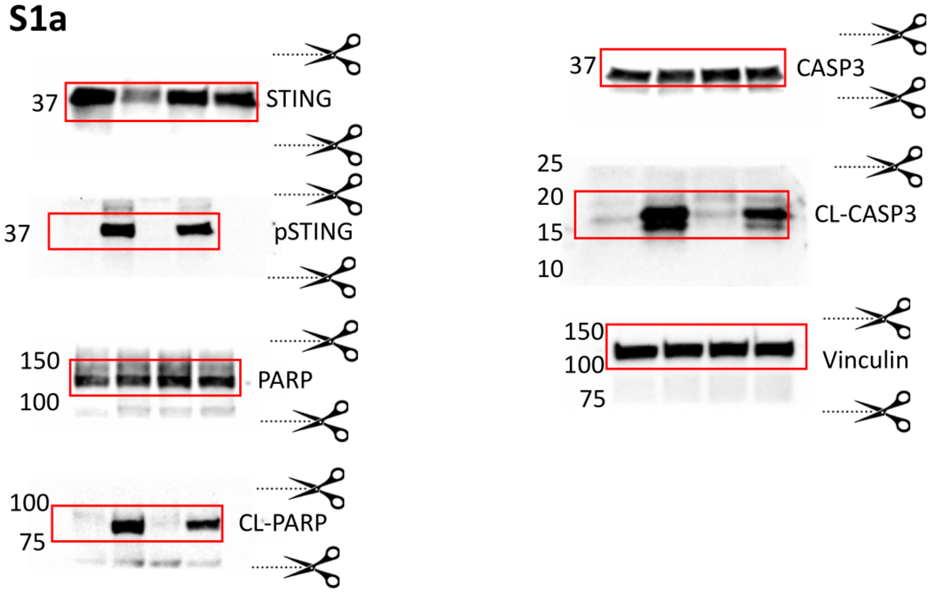

Fig. S1b

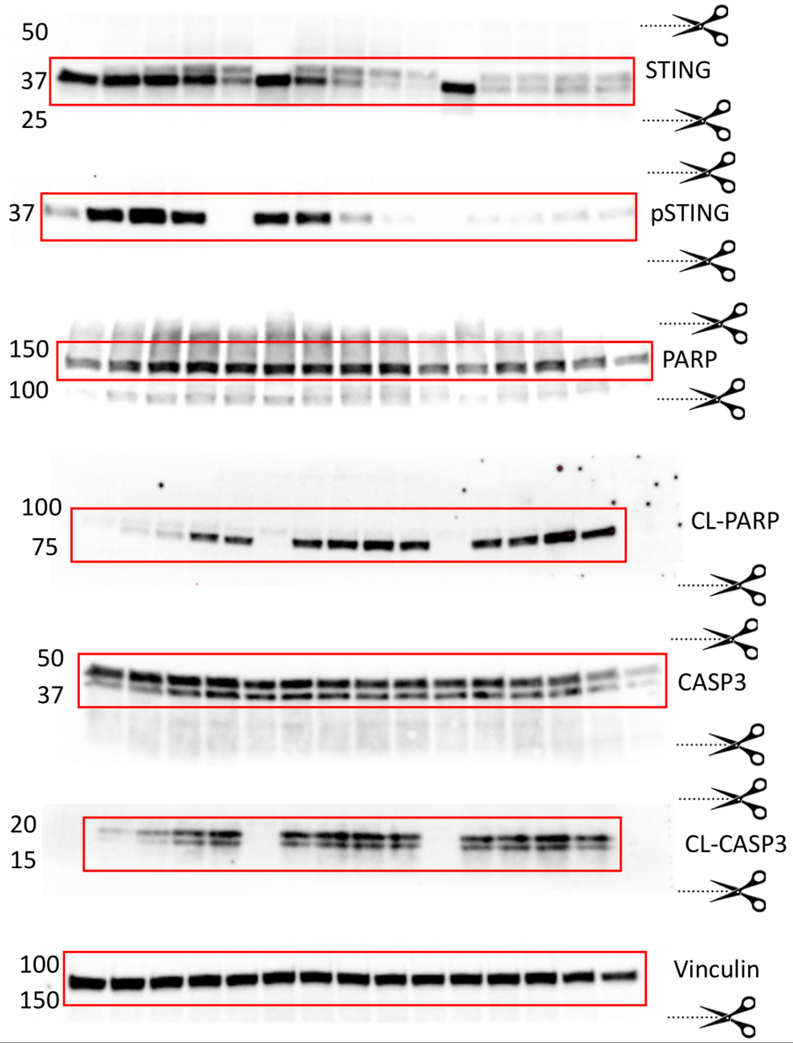

Fig. S2d

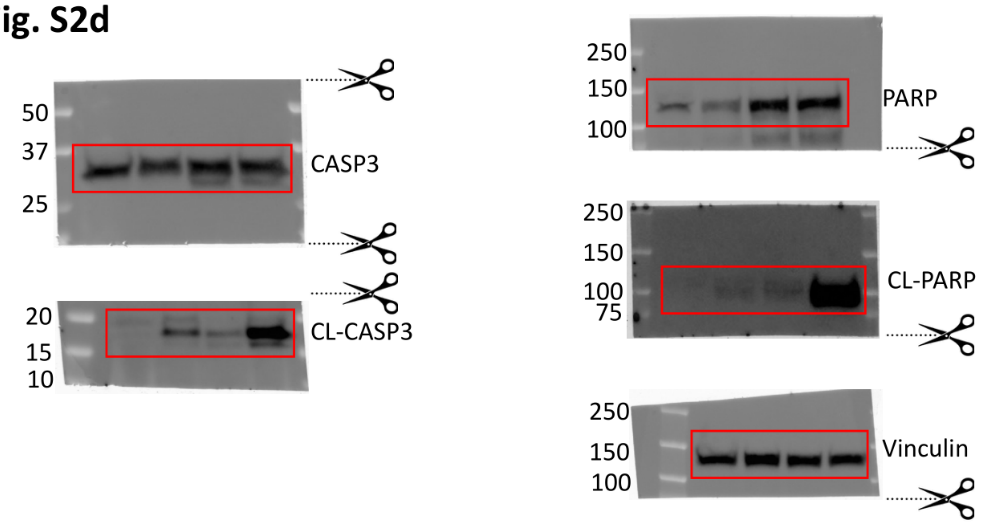

Fig. S2e

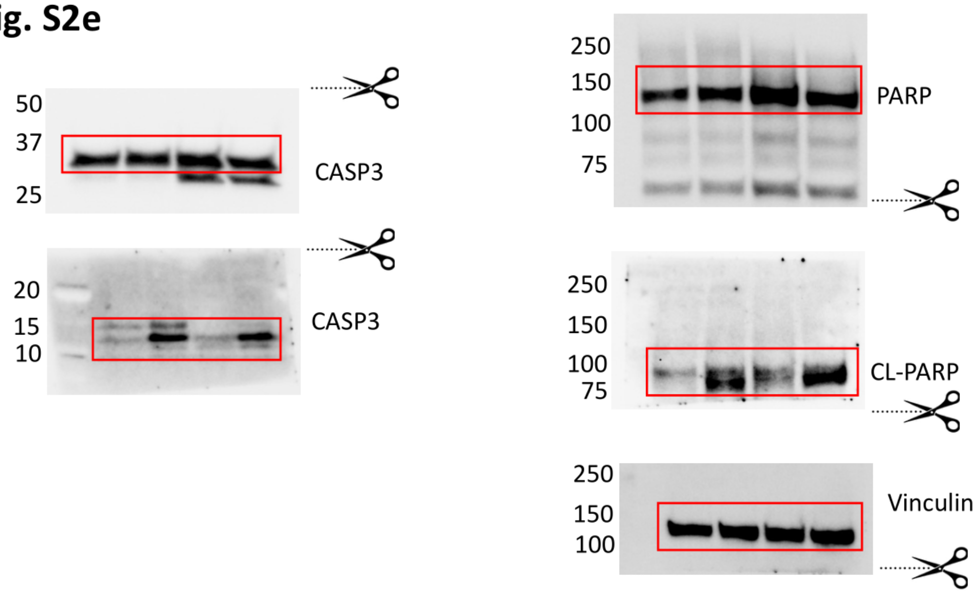

Fig. S2f

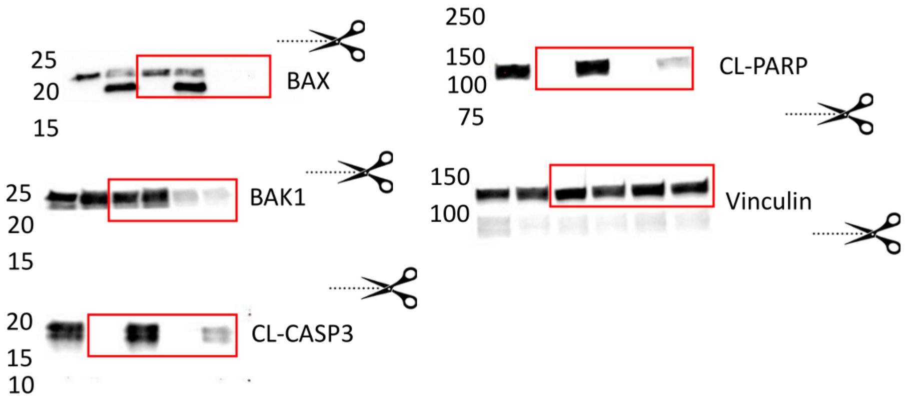

Fig. S2h

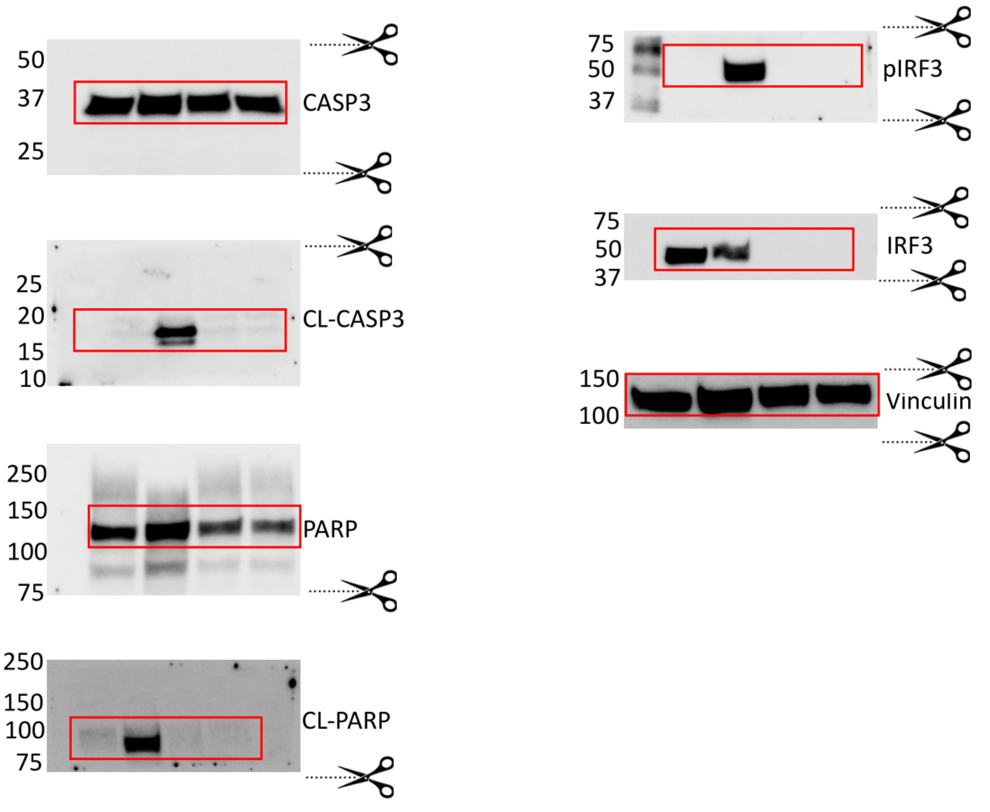

Fig. S2i

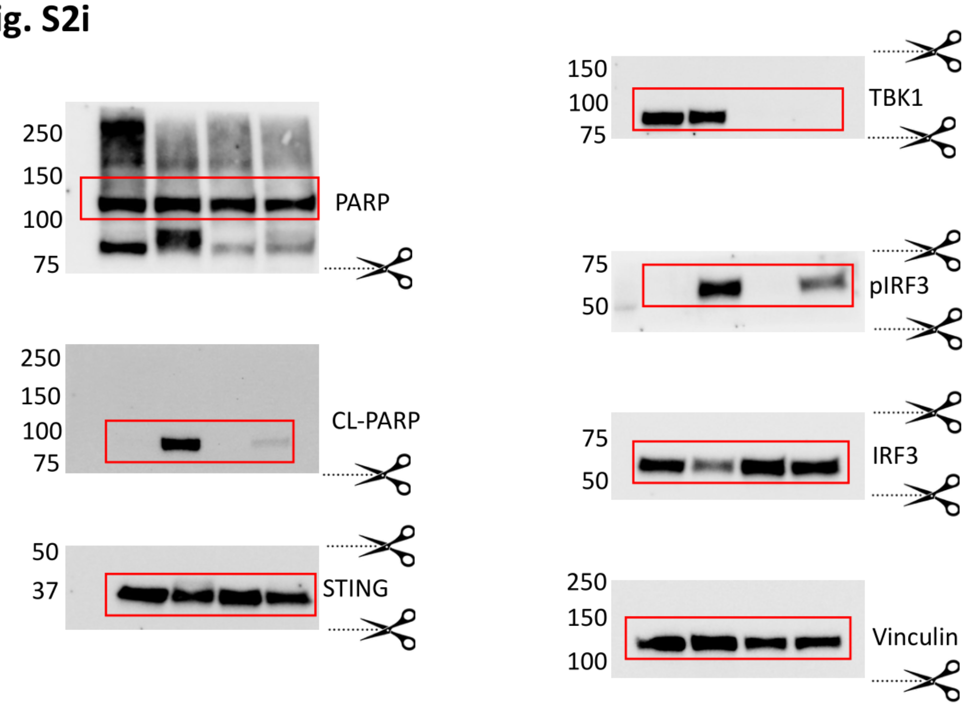

Fig. S4a

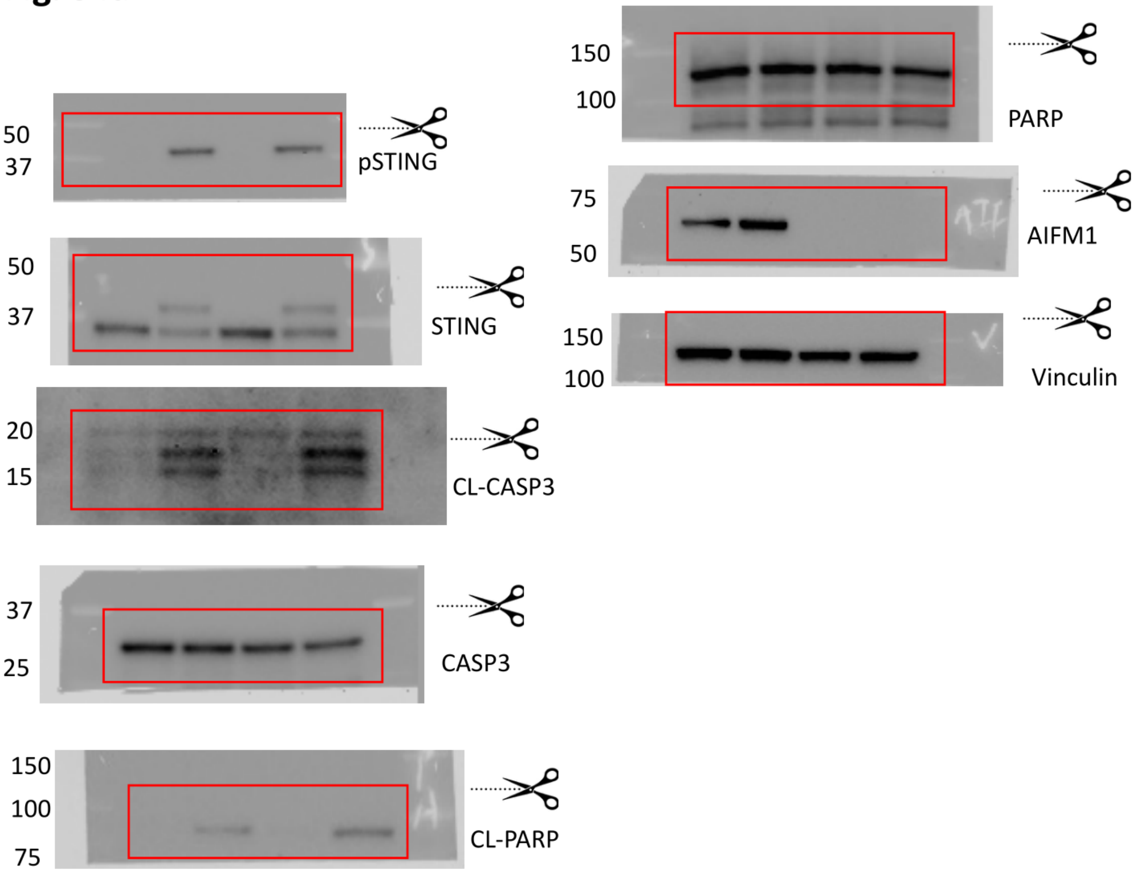

Fig. S4c

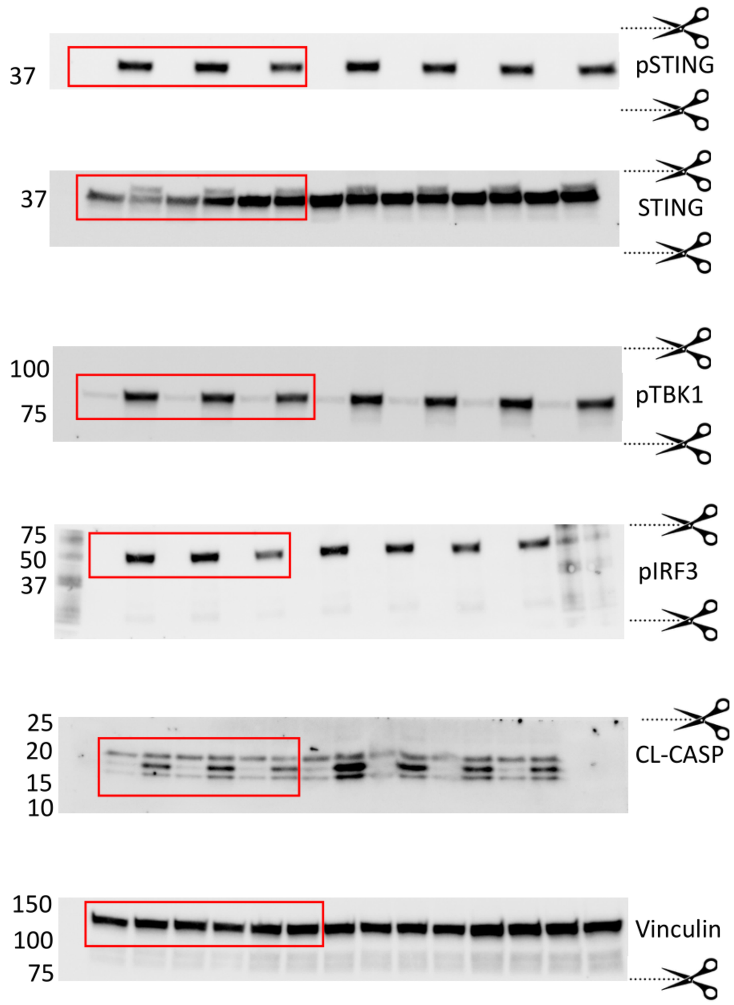
37
pSTING
37
STING
100
75
pTBK1
75
50
37
pIRF3
25
20
15
10
CL-CASP
150
100
75
Vinculin

Fig. S4d

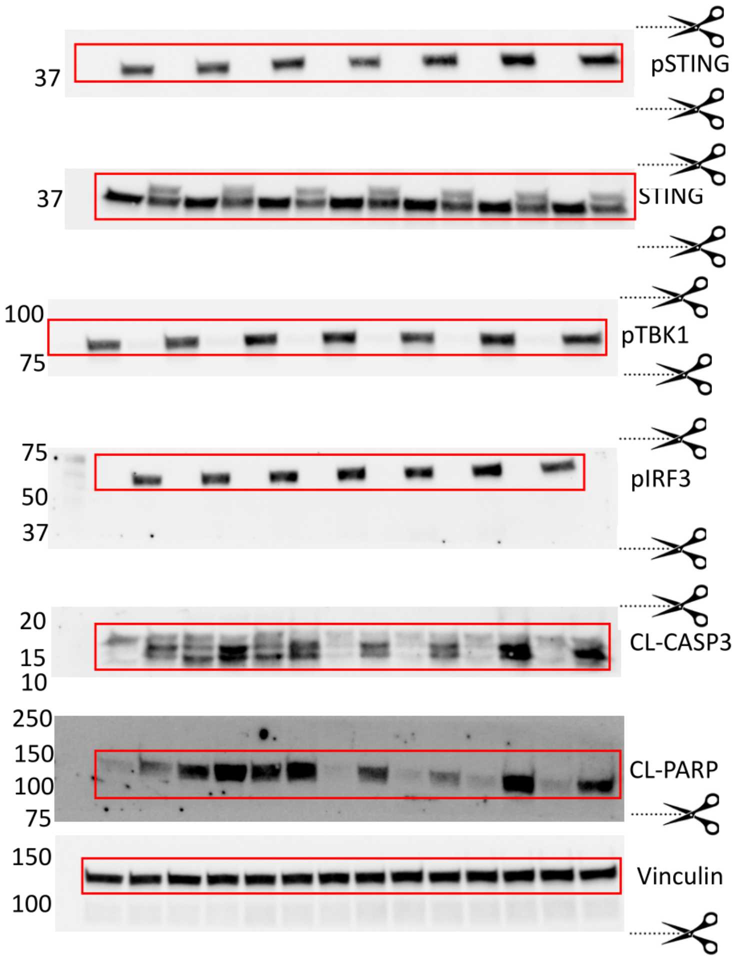
37
pSTING
37
STING
100
75
pTBK1
75
50
37
pIRF3
20
15
10
CL-CASP3
250
150
100
75
CL-PARP
150
100
Vinculin

Fig. S4e

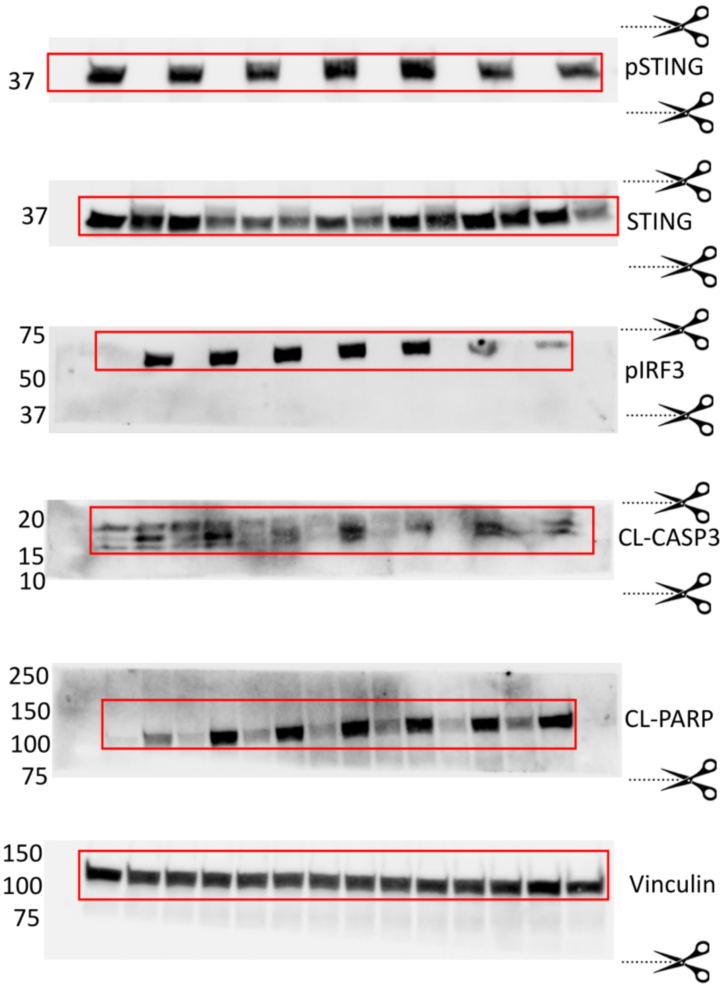

37
pSTING
37
STING
75
50
37
pIRF3
20
15
10
CL-CASP3
250
150
100
75
CL-PARP
150
100
75
Vinculin

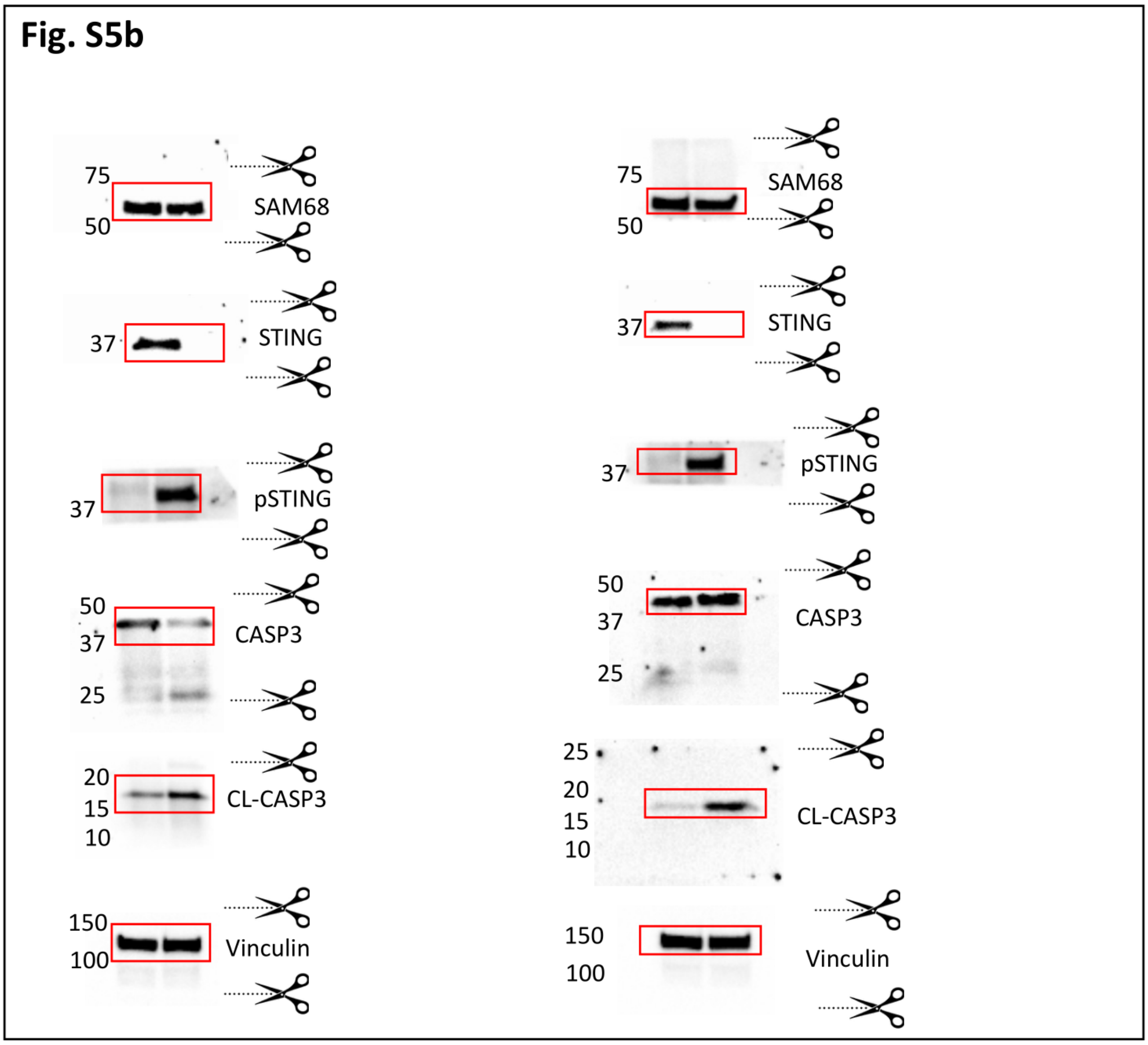
Fig. S5b
75
50
SAM68
37
STING
37
pSTING
50
37
25
CASP3
20
15
10
CL-CASP3
150
100
Vinculin
75
50
SAM68
37
STING
37
pSTING
50
37
25
CASP3
25
20
15
10
CL-CASP3
150
100
Vinculin

Fig. S5c

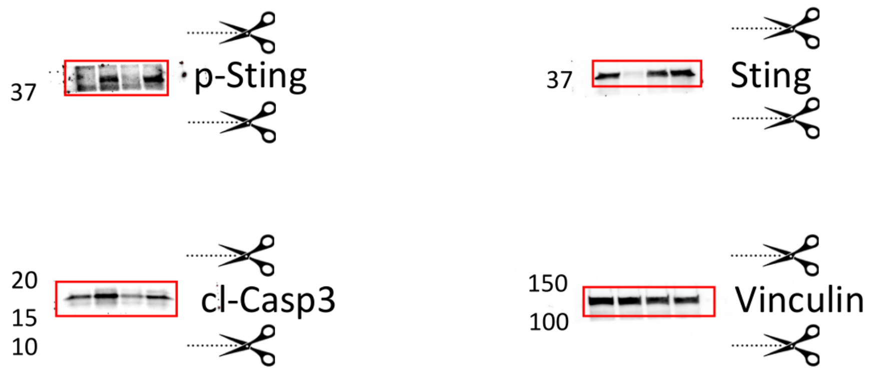

Fig. S5d

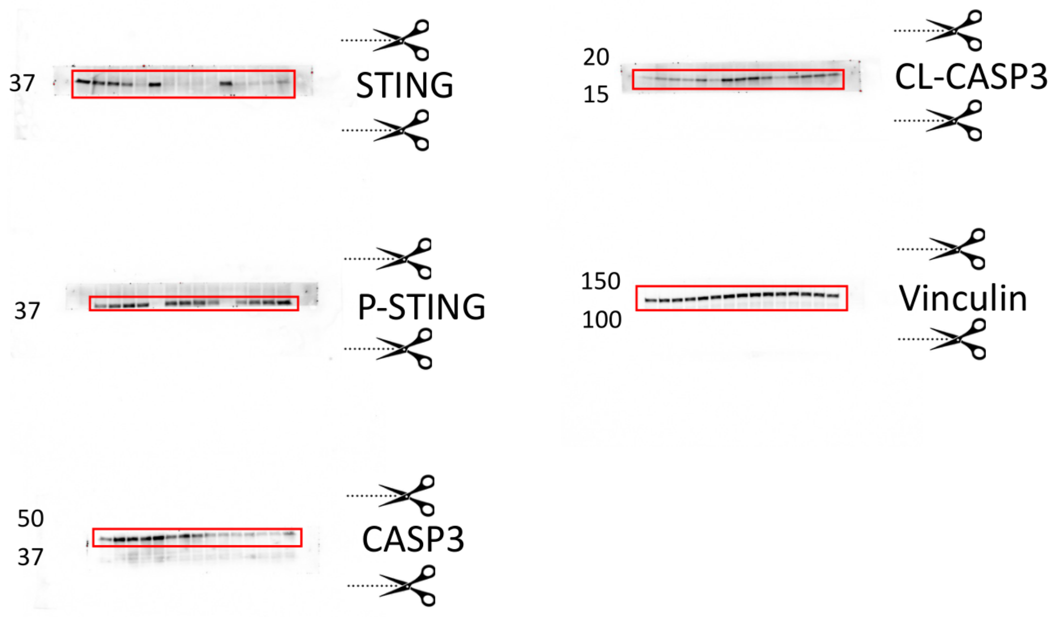

Fig. S6a

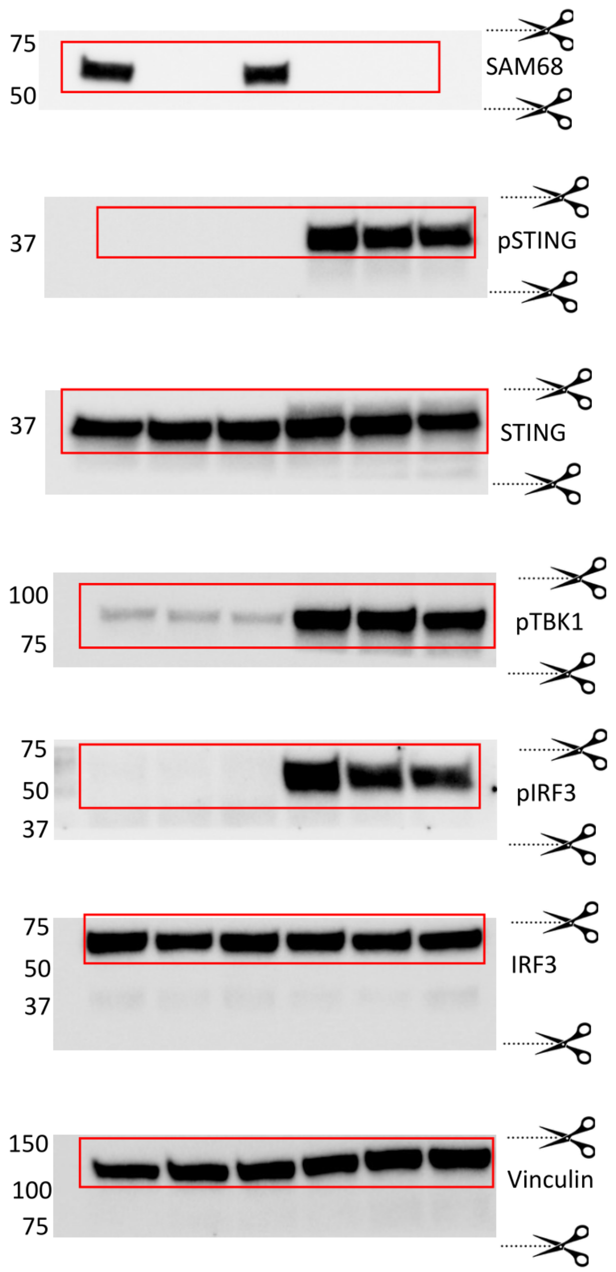
75
50
SAM68
37
pSTING
37
STING
100
75
pTBK1
75
50
37
pIRF3
75
50
37
IRF3
150
100
75
Vinculin

Fig. S7a

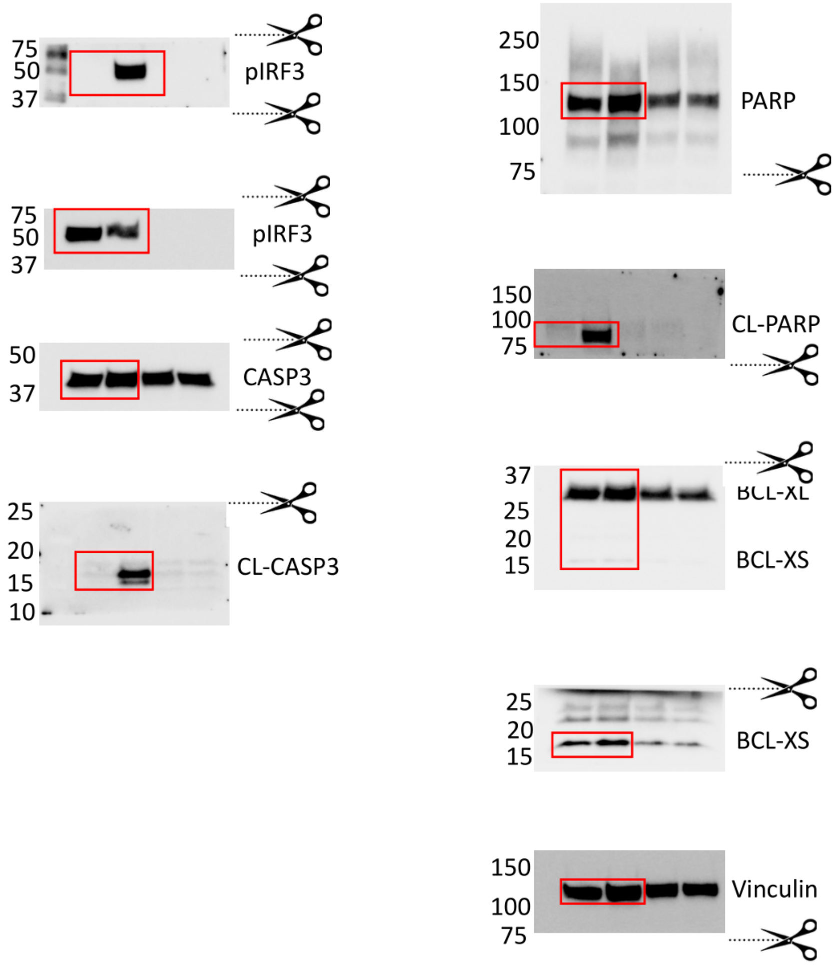
75
50
37
pIRF3
75
50
37
pIRF3
50
37
CASP3
25
20
15
10
CL-CASP3
250
150
100
75
PARP
150
100
75
CL-PARP
37
25
20
15
BCL-XL
BCL-XS
25
20
15
BCL-XS
150
100
75
Vinculin

Supplementary Data 1 (see supplementary data)

Mass spectrometry dataset on proteins co-immunoprecipitating with STING.

Supplementary Table S1

gRNA sequences used in the study.

Target	sgRNA sequence	Company
AAVS1	5'-G*G*G*GCCACUAGGGACAGGAU	Synthego
BAK1	5'-G*C*U*CACCUGCUAGGUUGCAG	Synthego
BAX	5'-C*C*C*ACAGGCCCUGUGCACCA	Synthego
AIMF1	5'-U*G*G*CUAGCUCUGGUGCAUCA	Synthego
DDX1 1	5'-G*C*U*GAACUGAAAUUUAACUU	Synthego
DDX1 2	5'-G*C*A*ACAAAGCCAUCUUUUGG	Synthego
RBM14 1	5'-C*G*A*CAAAAUGAAGAUAUUCG	Synthego
RBM14 2	5'-A*U*A*UUCGUGGGCAACGUCGA	Synthego
FAM98A 1	5'-A*C*G*UUUCGGCUGGUAAACCU	Synthego
FAM98A 2	5'-C*C*A*GACAGAAAAAUUAGCCA	Synthego
CAPRIN 1	5'-C*A*A*AAAAGAAGACAGCACGU	Synthego
CAPRIN 2	5'-A*A*U*UGGGAGAUGAUGAAGUG	Synthego
FAM98B 1	5'-A*G*A*AGAGCAAGCCCUUACAA	Synthego
FAM98B 2	5'-A*G*G*GCUUGCUCUUCUAACAA	Synthego
LARP1 1	5'-G*G*C*AGUUUACGGGUAGGCUG	Synthego
LARP1 2	5'-G*G*C*UGAGGCUUGUGGGACUG	Synthego
RALY 1	5'-U*G*C*CUGAAGCUUCAAGGACA	Synthego
RALY 2	5'-C*U*C*GAGAGUUGAUGGACUUG	Synthego
KHDRBS1 1	5'-G*A*A*AUUGAGAAGAUUCAGAA	Synthego
KHDRBS1 2	5'-A*G*A*CUCAAAAAAGGAUGAUG	synthego

Supplementary Table S2

Primers used in the study.

Gene	Forward primer 5'-3'	Reverse primer 5'-3'	Sequencing primer 5'-3'
DDX1 #1	GCGGATGGGTCTAGTCTA	GAGACGCCTTACTTACTGGAG	GAGACGCCTTACTTACTGGAG
DDX1 #2	GCGGATGGGTCTAGTCTA	CCC TGG CAC TGA ATA AGC AC	CCC TGG CAC TGA ATA AGC AC
RBM14	CTGAGGAGGACTGCCGGTC	CGCTTGAGCACATTCATCGG	CGT TAC CTT TCA CCA CGT CA
FAM98A	CACGTTGGCCTCATAACCCT	GACCTCCTCGTCCTCCGTAT	CACGTTGGCCTCATAACCCT
CAPRIN1 #1	TTGGGTCATTTGCCCATAGAGG	TCAAGCTCATGTCCCGTTCA	TCAAGCTCATGTCCCGTTCA
CAPRIN1 #2	TTGGGTCATTTGCCCATAGAGG	TTT CCT TCC CTT CCA GCA GG	TTT CCT TCC CTT CCA GCA GG
FAM98B	TTTGACTTTGTGATACCTGTGGT	TATCAGAGCCGTCAGGAAGAG	TTTGACTTTGTGATACCTGTGGT
LARP1	CGCTGTCCTGAAGGTTGTCT	CATGGCCACAGAATCCCCTT	CGCTGTCCTGAAGGTTGTCT
RALY	GCAGCCAATATGTGGCAACTC	CCTAAGTCATCTGTACCCGGTG	GCAGCCAATATGTGGCAACTC
KHDRBS1	AGAGTACTAATGTCACCGTGGA	CCCCAGGAGTCTTTATTATAGCC	AGAGTACTAATGTCACCGTGGA

Supplementary Table S3

Antibodies used in the study.

Primary Antibodies	Use	Species	Dilution	Company	Catalogue Number
anti Bcl-XL/XS	WB	Rabbit mAB	1:1.000	Cell Signaling	#2764
anti caspase 3	WB	Rabbit mAB	1:1.000	Cell Signaling	#9662
anti cGAS	WB	Rabbit mAB	1:1.000	Cell Signaling	#79978
anti cleaved caspase 3	WB	Rabbit mAB	1:1.000	Cell Signaling	#9664
anti cleaved PARP	WB	Rabbit mAB	1:1.000	Cell Signaling	#5625
anti COX IV	WB	Rabbit mAB	1:1.000	Abcam	#ab33985
anti cytochrome C	WB	Rabbit mAB	1:1.000	Cell Signaling	#11940
anti GM130	WB	Rabbit mAB	1:1.000	Cell Signaling	#12480
anti IFIT1	WB	Rabbit mAB	1:1.000	Cell Signaling	#14769
anti IRF3	WB	Rabbit mAB	1:1.000	Cell Signaling	#11904
anti ISG15	WB	Rabbit mAB	1:1.000	Cell Signaling	#2758
anti LC3BII	WB	Rabbit mAB	1:1.000	Cell Signaling	#3868
anti PARP	WB	Rabbit mAB	1:1.000	Cell Signaling	#9532
anti phospho IRF3 (Ser396)	WB	Rabbit mAB	1:1.000	Cell Signaling	#29047
anti phospho STAT1 (Tyr701)	WB	Rabbit mAB	1:1.000	Cell Signaling	#7649
anti phospho STING (S366)	WB	Rabbit mAB	1:1.000	Cell Signaling	#50907
anti phospho TBK1/NAK (Ser172)	WB	Rabbit mAB	1:1.000	Cell Signaling	#5483
anti SAM68	WB	Rabbit mAB	1:1.000	Cell Signaling	#12538
anti SEC61B	WB	Rabbit mAB	1:1.000	Cell Signaling	#16648
anti STING	WB	Rabbit mAB	1:1.000	Cell Signaling	#13647
anti TBK1/NAK	WB	Rabbit mAB	1:1.000	Cell Signaling	#3504
anti vinculin	WB	Mouse mAB	1:10.000	Sigma	#V9264
anti cleaved caspase 3	Confocal	Rabbit mAB	1:400	Cell Signaling	#9664
anti IFIT1	Confocal	Rabbit mAB	1:800	Cell Signaling	#14769
anti DKK	Confocal	Mouse mAB	1:500	Origene	#TA50011-100
anti Bak	WB	Rabbit mAB	1:1.000	Cell Signaling	#12105
anti Bax	WB	Rabbit mAB	1:1.000	Cell Signaling	#5023
anti phospho STING (S365)	WB	Rabbit mAB	1:1.000	Cell Signaling	#72971
anti SAM68	Confocal	Rabbit mAB	1 in 100	Cell Signaling	#33210
anti GM130 (Golgi)	Confocal	Mouse mAB	1 in 200	BD transduction laboratories	#610823
anti mitochondria	Confocal	Mouse mAB	1 in 100	Abcam	#ab92824
anti STING	Confocal	Sheep mAB	1 in 50	R&D systems	#5-001-A
**Secondary Antibodies**	**Use**	**Species**	**Dilution**	**Company**	**Catalogue Number**
HRP conjugated anti-mouse	WB	Donkey	1:10.000	Jackson Immuno Research	715-036-150
HRP conjugated anti-rabbit	WB	Donkey	1:10.000	Jackson Immuno Research	711-035-152
Alexa 555 conjugated anti-mouse	Confocal	Goat	1:500	Invitrogen	A21424
Alexa 568 conjugated anti-sheep	Confocal	Donkey	1:500	Invitrogen	A21099
Alexa 568 conjugated anti-rabbit	Confocal	Donkey	1:500	Invitrogen	A10042
Alexa 488 conjugated anti-mouse	Confocal	Donkey	1:500	Invitrogen	A21141
Alexa 488 conjugated anti-sheep	Confocal	Donkey	1:500	Invitrogen	A11015
Alexa 488 conjugated anti-rabbit	Confocal	Goat	1:400	Invitrogen	A11008
Alexa 647 conjugated anti-mouse	Confocal	Donkey	1:500	Invitrogen	A31571
Alexa 647 Phalloidin	Confocal	x	1:400	Invitrogen	A30107
DAPI	Confocal	x	1:100	BioRad	1351303
