# Supplementary material for: SAM68 directs STING signaling to apoptosis in macrophages
Source: Commun Biol. 2024 Mar 7;7:283. doi: 10.1038/s42003-024-05969-1 (PMC10920828; doi:10.1038/s42003-024-05969-1)
Supplement: Supplementary file 3 — Description of Supplementary Materials [file 42003_2024_5969_MOESM3_ESM.docx]

**Description of Additional Supplementary Files**

File name: Supplementary Data 1

Description: Mass spectrometry dataset on proteins co-immunoprecipitating with STING

File name: Supplementary Data 2

Description: The source data behind the graphs in the paper
