# Supplementary material for: SAM68 directs STING signaling to apoptosis in macrophages
Source: Commun Biol. 2024 Mar 7;7:283. doi: 10.1038/s42003-024-05969-1 (PMC10920828; doi:10.1038/s42003-024-05969-1)
Supplement: Supplementary file 6 — Reporting Summary [file 42003_2024_5969_MOESM6_ESM.pdf]

nature portfolio

Corresponding author(s): David OLAGNIER

Last updated by author(s): Feb 1, 2024

Reporting Summary

Nature Portfolio wishes to improve the reproducibility of the work that we publish. This form provides structure for consistency and transparency in reporting. For further information on Nature Portfolio policies, see our Editorial Policies and the Editorial Policy Checklist.

Statistics

For all statistical analyses, confirm that the following items are present in the figure legend, table legend, main text, or Methods section.

n/a	Confirmed	
☐	☒	The exact sample size (*n*) for each experimental group/condition, given as a discrete number and unit of measurement
☐	☒	A statement on whether measurements were taken from distinct samples or whether the same sample was measured repeatedly
☐	☒	The statistical test(s) used AND whether they are one- or two-sided<br>*Only common tests should be described solely by name; describe more complex techniques in the Methods section.*
☒	☐	A description of all covariates tested
☐	☒	A description of any assumptions or corrections, such as tests of normality and adjustment for multiple comparisons
☐	☒	A full description of the statistical parameters including central tendency (e.g. means) or other basic estimates (e.g. regression coefficient) AND variation (e.g. standard deviation) or associated estimates of uncertainty (e.g. confidence intervals)
☒	☐	For null hypothesis testing, the test statistic (e.g. *F*, *t*, *r*) with confidence intervals, effect sizes, degrees of freedom and *P* value noted<br>*Give P values as exact values whenever suitable.*
☒	☐	For Bayesian analysis, information on the choice of priors and Markov chain Monte Carlo settings
☒	☐	For hierarchical and complex designs, identification of the appropriate level for tests and full reporting of outcomes
☒	☐	Estimates of effect sizes (e.g. Cohen's *d*, Pearson's *r*), indicating how they were calculated

Our web collection on statistics for biologists contains articles on many of the points above.

Software and code

Policy information about availability of computer code

Data collection	not applicable
Data analysis	not applicable

For manuscripts utilizing custom algorithms or software that are central to the research but not yet described in published literature, software must be made available to editors and reviewers. We strongly encourage code deposition in a community repository (e.g. GitHub). See the Nature Portfolio guidelines for submitting code & software for further information.

Data

Policy information about availability of data

All manuscripts must include a data availability statement. This statement should provide the following information, where applicable:

- Accession codes, unique identifiers, or web links for publicly available datasets
- A description of any restrictions on data availability
- For clinical datasets or third party data, please ensure that the statement adheres to our policy

The data that support the findings of this study are available from the corresponding author upon request.

Human research participants

Policy information about studies involving human research participants and Sex and Gender in Research.

Reporting on sex and gender	not applicable
Population characteristics	not applicable
Recruitment	not applicable
Ethics oversight	not applicable

Note that full information on the approval of the study protocol must also be provided in the manuscript.

Field-specific reporting

Please select the one below that is the best fit for your research. If you are not sure, read the appropriate sections before making your selection.

☒ Life sciences ☐ Behavioural & social sciences ☐ Ecological, evolutionary & environmental sciences

For a reference copy of the document with all sections, see nature.com/documents/nr-reporting-summary-flat.pdf

Life sciences study design

All studies must disclose on these points even when the disclosure is negative.

Sample size	The data are shown as means of biological replicates +/- s.e.m. If indicated, data were analyzed for statistical significant differences between groups using a two-tailed unpaired Student's t-test (** p < 0.01; *** p < 0.001;**** p < 0.0001) or a two-tailed one-way ANOVA followed by Sidak's multiple comparison test (** p < 0.01; *** p < 0.001;**** p < 0.0001).
Data exclusions	not applicable
Replication	The data are the means of 2-4 independent experiments performed in 3-6 biological replicates unless otherwise mentioned.
Randomization	not applicable
Blinding	not applicable

Reporting for specific materials, systems and methods

We require information from authors about some types of materials, experimental systems and methods used in many studies. Here, indicate whether each material, system or method listed is relevant to your study. If you are not sure if a list item applies to your research, read the appropriate section before selecting a response.

Materials & experimental systems

n/a	Involved in the study	
☐	☒	Antibodies
☐	☒	Eukaryotic cell lines
☒	☐	Palaeontology and archaeology
☐	☒	Animals and other organisms
☒	☐	Clinical data
☒	☐	Dual use research of concern

Methods

n/a	Involved in the study	
☒	☐	ChIP-seq
☐	☒	Flow cytometry
☒	☐	MRI-based neuroimaging

Antibodies

Antibodies used	Information is provided in Supplementary Table 3
Validation	Not all antibodies used in our study were validated. Some were when KO cell lines were used

Eukaryotic cell lines

Policy information about cell lines and Sex and Gender in Research

Cell line source(s)	WT THP1 cells (ATCC)<br>THP1-DualTM cells, THP1-DualTM KO-cGAS cells, THP1-DualTM KO-STING cells, THP1-DualTM KO-IRF3 cells, THP1-DualTM KO-TBK1 cells, THP1-DualTM KO-IFNAR2 cells, THP1-DualTM KO-ASC cells (Invivogen)<br>Monocytes were isolated from PBMCs isolated from buffy coats from the Aarhus University Hospital blood bank.
Authentication	None of the cell lines were authenticated. Most of the cell lines were ordered from ATCC or Invivogen
Mycoplasma contamination	All cells were tested negative for mycoplasma contamination.
Commonly misidentified lines (See ICLAC register)	We did not use misidentified cell lines in this study

Animals and other research organisms

Policy information about studies involving animals; ARRIVE guidelines recommended for reporting animal research, and Sex and Gender in Research

Laboratory animals	WT and SAM68 KO mice were on a C57BL/6J genetic background and were between 8-12 week old.<br><br>Animals dedicated for bone marrow extraction were housed at Aarhus University, Department of Biomedicine, under license 2023-15-0201-01489. Bone marrow was extracted from the femur and tibia of 10-14-week-old female C57BL/6BomTac mice
Wild animals	Wild animals were not used in this study
Reporting on sex	no sex report on this study
Field-collected samples	no samples were collected from the field in this study
Ethics oversight	Mice breeding, housing and treatments were conducted according to the Guidelines of the Italian Institute of Health approved by the protocol n. 157/2019-PR<br><br>Animals dedicated for bone marrow extraction were housed at Aarhus University, Department of Biomedicine, under license 2023-15-0201-01489.

Note that full information on the approval of the study protocol must also be provided in the manuscript.

Flow Cytometry

Plots

Confirm that:

- [ ] The axis labels state the marker and fluorochrome used (e.g. CD4-FITC).
- [ ] The axis scales are clearly visible. Include numbers along axes only for bottom left plot of group (a 'group' is an analysis of identical markers).
- [ ] All plots are contour plots with outliers or pseudocolor plots.
- [x] A numerical value for number of cells or percentage (with statistics) is provided.

Methodology

Sample preparation	The Dead Cell Apoptosis Kit with Annexin V FITC and PI (Invitrogen) was used to detect apoptosis by flow cytometry. Apoptosis was induced by dsDNA transfection or cGAMP stimulation as described above. Untreated cells served as a negative control. Harvested cells were washed in cold PBS and resuspended in 1x Annexin-binding buffer. To stain the cells, 5 µl of AlexaFluor488 Annexin-V and 1 µl of 100 µg/ml PI solution were added per 100 µl of cell suspension and incubated for 15 minutes at RT.
Instrument	NovoCyte Flow Cytometer (ACEA Biosciences)
Software	FlowJo software v10.6.2 (FlowJo LLC)
Cell population abundance	N/A

Gating strategy

Gating strategy is displayed in the supplementary material

☒ Tick this box to confirm that a figure exemplifying the gating strategy is provided in the Supplementary Information.
